# Population genetics and adaptation to climate along elevation gradients in invasive *Solidago canadensis*

**DOI:** 10.1371/journal.pone.0185539

**Published:** 2017-09-28

**Authors:** Emily V. Moran, Andrea Reid, Jonathan M. Levine

**Affiliations:** 1 Life and Environmental Sciences, University of California Merced, Merced, CA, United States of America; 2 Biology, ETH Zürich, Zürich, Switzerland; National Cheng Kung University, TAIWAN

## Abstract

Gene flow between populations may either support local adaptation by supplying genetic variation on which selection may act, or counteract it if maladapted alleles arrive faster than can be purged by selection. Although both such effects have been documented within plant species’ native ranges, how the balance of these forces influences local adaptation in invasive plant populations is less clear, in part because introduced species often have lower genetic variation initially but also tend to have good dispersal abilities. To evaluate the extent of gene flow and adaptation to local climate in invasive populations of *Solidago canadensis*, and the implications of this for range expansion, we compared population differentiation at microsatellite and chloroplast loci for populations across Switzerland and assessed the effect of environmental transfer distance using common gardens. We found that while patterns of differentiation at neutral genetic markers suggested that populations are connected through extensive pollen and seed movement, common-garden plants nonetheless exhibited modest adaptation to local climate conditions. Growth rate and flower production declined with climatic distance from a plant's home site, with clones from colder home sites performing better at or above the range limit. Such adaptation in invasive species is likely to promote further spread, particularly under climate change, as the genotypes positioned near the range edge may be best able to take advantage of lengthening growing seasons to expand the range.

## Introduction

Local adaptation is a central process determining the limits to species’ ranges and how they respond to environmental change. The relationship between gene flow and adaptation to local conditions is complex [1–4]. High levels of gene flow between environmentally distinct areas can inhibit adaptation and contribute to range boundaries by “swamping” small peripheral populations with maladapted alleles [5–8]. According to theory, such effects are most likely when environmental gradients are steep (enabling higher gene flow) and fitness effects of key traits contrast at different points along the gradient, or when the strength or effectiveness of selection is weak (eg. in small populations where drift dominates)[2]. Alternatively, gene flow may enhance adaptation by increasing local genetic diversity, an important prerequisite for a population to respond to natural selection [9–13]. In such cases, the beneficial effects of immigration on genetic variation and population size, including the alleviation of inbreeding and drift, outweigh the swamping effects of arriving alleles [2,8,10,14,15]. There is empirical evidence for both effects of gene flow from studies of species native ranges [1,3,16–18], though positive effects of gene flow have most often been observed in experimental evolution studies [1,2,19].

Strong local adaptation despite high pollen exchange is also observed where deleterious non-local alleles are purged by strong selection [2,20]. This results in "isolation by environment" (IBE) at ecologically important loci, and sometimes at linked neutral loci. In a recent review, Sexton et al. [8] found that, out of 26 studies on plants, 30.8% detected only classic isolation-by-distance (IBD), 19.2% identified IBE, 38.5% found both IBE and IBD, and 19.2% showed counter-gradient gene flow (with higher gene flow between disparate environments). More generally, theory suggests that the strength of selection is a key determinant of whether gene flow benefits or harms local adaptation [8,14,15].

Better understanding adaptation in the presence of gene flow is particularly important for forecasting the spread of invasive species under current and future climates [21,22]. As invasive species expand their range, they encounter new environments, and thus their spread may be intimately tied to their ability to adapt to local conditions. Adaptation to growing season length, for example, has been demonstrated to have facilitated the northward spread of *Lythrum salicaria* [23]. Moreover, adaptation in invasive species along local climate gradients could facilitate expansion beyond the current range edge. This would be expected if plants near the range edge have the traits necessary for positive population growth beyond that edge with a modest shift in trait values or environmental conditions, because the furthest-forward individuals in a population tend to play an important role in enabling population spread [24,25].

There are reasons to expect that how gene flow affects local adaptation may differ for invasive versus native species. First, introduced species are often subject to genetic bottlenecks, which reduce overall genetic diversity, and potentially reduce adaptive potential [21,26]. Thus, introduced populations may benefit more than native populations from high gene flow, especially if this boosts local variation in ecologically important traits. Admixture between populations has been documented to boost fitness or invasion success, particularly for populations suffering from inbreeding depression [27–29]. On the other hand, some invaders achieve success despite low genetic diversity [30], and some of these have exhibited adaptation to local conditions in the new range [31]. Because many invasive species have traits that tend to promote dispersal and gene flow such as the production of many small seeds [32,33], and benefit from more frequent human-assisted transport, gene flow between populations is likely to be particularly high. However, due to a paucity of empirical studies in introduced populations, we currently lack clear expectations about how gene flow influences the range limits of invasive populations, including whether it is likely to help or hinder expansion beyond the current range limits [22].

Here, we explore gene flow, adaptation to local climate, and performance within and beyond the current elevational range limit in Swiss populations of *Solidago canadensis*, a species native to North America and invasive in Europe and Asia. Previously observed genetic differences in plant size and phenology along latitudinal gradients in Europe are consistent with adaptation in the introduced range [34]. Nonetheless, because *S*. *canadensis* has small wind-dispersed seeds and has also been transported by humans, gene flow may be extensive enough to impede adaptation to local conditions over the steep environmental gradients in mountain environments.

In this study, we ask the following three questions:

1. Do populations of *S*. *canadensis* exhibit genetic structure or isolation-by-distance (IBD) consistent with limited seed or pollen dispersal, or isolation-by-environment (IBE) consistent with selection against immigrants or biased dispersal between environments?
2. Does *S*. *canadensis* exhibit adaptation along climate gradients, exhibiting higher performance in environments more climatically similar to their home?
3. Do individuals from higher elevation perform better beyond the current high-elevation range limit than their lower elevation counterparts?

We sampled 43 populations from across Switzerland to estimate genetic diversity and population structure, genotyping all individuals at 7 nuclear microsatellite (hereafter SSR) loci and sequencing 4 chloroplast intergenic regions in a subset of individuals. We then examined the performance of clones from 13 populations differing in home elevation in three common gardens positioned along a steep elevation gradient.

## Methods

### The study species

We refer to our study species as *Solidago canadensis* following the most common naming convention in Switzerland and surrounding countries today. However, this species is referred to as *S*. *altissima* in earlier literature [35], and there is still some debate surrounding the proper taxonomy of the genus [36–38].

Regardless of the nomenclature, *S*. *canadensis sensu lato* was introduced to London in 1738 [34], and from there was transported across Europe [39]. It has since spread extensively in disturbed areas such as roadsides, and is considered invasive. It was introduced to Switzerland in 1850 [40], and blacklisted for sale there in 2012. There is no historical evidence of multiple introductions [34], and low microsatellite allelic richness in European versus native range populations is consistent with a genetic bottleneck [41]. *S*. *gigantea* is also invasive in Switzerland, but can be easily distinguished by morphology and habitat [39], as well as ploidy level: European *S*. *gigantea* are tetraploid [42] while European *S*. *canadensis* is diploid.

*Solidago* species are insect-pollinated, self-incompatible perennials [39,43]. A seedling can grow to a clump of over 20 unbranched shoots (ramets) within 10 years [44]. For plants grown from rhizome fragments the “first year” number of ramets can be much higher, as our results here show. Shoots are annual, and regrow each spring. Typically, one inflorescence forms at the top of each shoot that reaches a sufficient size; each inflorescence can produce 20,000 wind-dispersed seeds [45]. As *Solidago* is autumn-blooming, one climatic constraint is the need to complete seed production before winter weather kills stems. In the native range, variation in flowering time was found to be heritable [46]. In both the native and invasive ranges of *S*. *canadensis*, reproduction from seed is crucial for the colonization of new sites. Disturbance is needed for this to occur, because seedlings cannot compete with established vegetation [44,47]. Once a population is established, the number of clones drops as individuals compete for space [44]. Rhizomes are relatively short in *S*. *canadensis/altissima* (<20 cm), so vegetative growth leads to diameter expansion rather than extensive spatial spread [45,48].

Before 1970, there were no recorded populations of *S*. *canadensis* in Switzerland above 700 m in elevation [40]. Today, virtually all populations occur below 1200 m [49], though there are a few records between 1200 and 1650 m [40]. The distances between populations increase with elevation [49]. The frequency of occurrences increased noticeably between 2008 and 2012 at low- to mid-elevations in the canton of Grisons, but the upper range boundary did not shift [49]. Similar patterns of decreasing probability of occurrence with elevation and relatively current stable range limits have been observed in multiple invasive species in montane environments [50].

### Population genetic sampling

**Fig 1:**
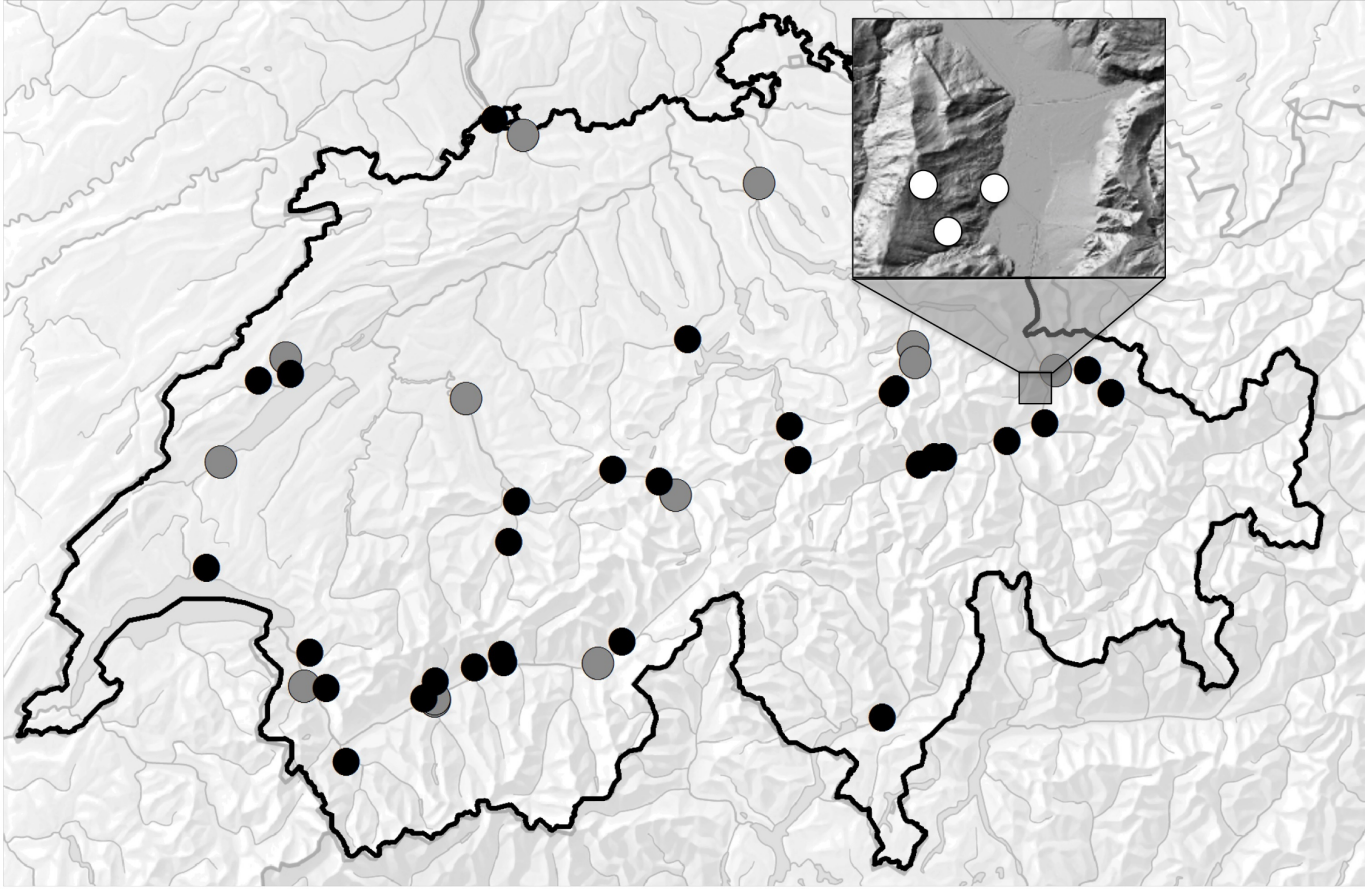
Map of sampled populations and common garden locations. Grey dots—populations included in population genetic analyses and common gardens. Black dots—populations included in population genetic analyses only. White dots in square inset—Common gardens.

To evaluate population structure and infer the degree of seed and pollen movement, we sampled 43 *S*. *canadensis* populations from across Switzerland in 2012, aiming to cover a wide range of elevations and human population densities (Fig 1; S1 Table; Dryad, doi:10.5061/dryad.h9r01). Sampled "populations" were separated by at least 490 m from each other and at least 200 m from unsampled patches of *S*. *canadensis*. However, this does not mean that we expected patches 200–490 m apart to be strongly reproductively isolated. Given the extensive travel distances possible for insect pollinators [51] and small wind-dispersed seeds [52], some minimum distance between sampled patches was desired to ensure that they were not too freely interbreeding. On the other hand, given the rapid change in climate with elevation in the mountains the minimum sampling distance should not be too long or it might have missed environmentally-driven differentiation: 490 m represented a compromise. For populations with 5–11 individuals, we collected leaf samples from all individuals, whereas if there were 12 or more individuals present we collected leaf samples from 11–19 haphazardly sampled individuals. To reduce the probability of sampling the same clone more than once, only clumps that were at least 1 m apart were sampled. Rhizome samples were collected from 5 individuals per population for propagation (see *Common gardens* below). Leaf samples were dried in labeled tea pouches over silica gel and genomic DNA was extracted following a modified CTAB protocol [53]. Multi-locus SSR genotypes of individuals within each population were compared, and one individual from any pair of duplicates (15 pairs from 12 populations) was removed so that only distinct genetic individuals were included in the population genetics calculations.

We tested 12 sets (forward/reverse) of SSR primers [54,55], and selected 7 that amplified correctly and had a low frequency of null alleles: SS1B, SS4G, SS19C, SS20E, SS4F, SS24F, and SC40 (Dryad, doi:10.5061/dryad.h9r01). All sampled individuals were genotyped at these loci. Details about primers tested and PCR protocols can be found in S1 File. While SSR marker variation reflects the movement of both seed and pollen, chloroplast haplotype variation reflects only the dispersal of seed or vegetative reproduction, as chloroplasts are maternally inherited. This contrast is useful because while both seed/rhizome and pollen dispersal can spread alleles between populations only dispersal of seed or rhizomes will result in population spread. We therefore also sequenced 215 individuals (5 per population) at four chloroplast DNA intergenic regions: trnV-atpE, trnK-rps16, trnH-psbA, and rpl36-rps8 [42,56]. Details of PCR protocols, sequencing, and alignment can be found in S2 File, along with GenBank numbers of the sequences.

### Measures of genetic differentiation

To evaluate IBD based on the SSR markers, we calculated both G_ST_ (multi-locus F_ST_) and D [57]—a measure that performs better for markers with high allelic richness—using Genalex [58]. Differentiation was calculated over all populations and between all pairs of populations. We examined the relationship between G_ST_ or D and distance class (10 km increments). We then used Mantel tests and created Mantel correlograms to test whether genetic differentiation is correlated with distance, and how this correlation changes with distance class (15 divisions). However, such a correlation does not in and of itself indicate IBD [59], as a relationship between geographic and genetic distance could occur due to environmental selection (resulting in IBE) if more distant locations are also more environmentally distinct. Therefore, we compared IBE to IBD using a multiple matrix regression approach (the R package MMRR [60]), where the matrix of pairwise differentiation measures is a function of both geographic and environmental distance between the populations. For the IBE versus IBD analysis, values in the environmental distance matrices were calculated as d_ij_ = d_ji_ = |X_i—_X_j_|, where X_i_ and X_j_ are the environmental variables described in the next section for populations *i* and *j*, respectively. However, because SSR markers are usually neutral, we did not expect to see a pattern of IBE unless at least one marker is closely linked to a gene under selection. Finally, we performed the Rousett test of IBD: if the stepping-stone model of IBD holds, G_ST_/(1-G_ST_) should increase linearly with the natural log of distance [61]. For all these analyses, we examined geographic distance-by-road (Dryad, doi:10.5061/dryad.h9r01) in addition to straight-line geographic distance between populations. Straight-line distances often crossed elevations >1600 m, across which seeds and pollinators are unlikely to move, and *Solidago* populations also tend to be concentrated near roads and railways.

For chloroplast markers, we computed differentiation between populations based on haplotypes ordered by similarity (N_ST_; [62]) and unordered haplotypes (G_ST_;[63]). If N_ST_ is significantly greater than G_ST_, this indicates spatial phylogenetic structure in chloroplast diversity, as one would expect for populations that diversified as they spread. These analyses were carried out in the program Permut, using 1000 permutations (https://www6.bordeaux-aquitaine.inra.fr/biogeco/Production-scientifique/Logiciels/Contrib-Permut/Permut). Finally, we estimated the ratio of seed to pollen movement using the following formula:

mpms=2(1GSTC−1)−(1GSTN−1)(1GSTC−1)

where m_p_ is pollen movement, m_s_ is seed movement, G_STC_ is chloroplast G_ST_ and G_STN_ is nuclear marker G_ST_ [64]_._ The formula was derived to reflect the fact that because nuclear markers like SSRs travel with both seed and pollen, the degree of differentiation at chloroplast loci is expected to be higher than differentiation at nuclear markers even if seed and pollen movement are equal. For this analysis, we calculated G_STN_ using the SSR genotypes of the same 5 individuals per population as were sequenced at the chloroplast markers.

### Climate data

To calculate climatic variables for source populations, we obtained climate data interpolated from ca. 400 Swiss climate stations (Bundesamt für Meteorologie und Klimatologie MeteoSchweiz). The climate data were based on a 30 year average (1961–1990) and were spatially interpolated using DayMet [65] for all sampled populations and the three common gardens described below. These data included average degree-days per year above 5.56°C (a base temperature chosen as a compromise between values from 3–7 degrees used in the plant literature [66–68]), average July temperature (°C), an index of growing season frost events [69], average days of precipitation June-August, average monthly precipitation (cm), and average global potential shortwave radiation for March and over the full year for 1961–1990 (KJ/m^2^/day). To account for topographical properties of the sites, we also included slope and aspect from a 25 m DEM (DHM25 (c) 1994 Bundesamt für Landestopographie). Data processing was performed at Landschaftsdynamik, WSL, and data are available on Dryad (doi:10.5061/dryad.h9r01). To make the scale of the variables more similar and aid comparison of effect sizes, we divided degree-days by 100 and both potential shortwave radiation measures by 1000.

Environmental distances between populations were calculated as d_ij_ = X_i—_X_j_, where X_i_ and X_j_ are the environmental variables of the planting site and home site, respectively. A negative degree-day distance, for example, means that a clone was planted at a site colder than the home site. We calculated an overall environmental distance using PCA, with ED_ij_ corresponding to the Euclidian distance between i and j based on PCA1 & PCA2. The two solar radiation measures were highly correlated with one another, as were the total precipitation with precipitation frequency and July temperature with degree-days.

### Common garden experiment

To evaluate whether populations showed adaptation to climate gradients, we grew clonal individuals in experimental gardens at three elevations in the Alps. This experiment tested “adaptation to climate” as opposed to “local adaptation” because we did not reciprocally transplant individuals between populations. Rather, as is common practice in tree provenance trials [70,71], individuals from different environments were planted in gardens differing in climate, in this case located along an elevation gradient. The principles are similar in both approaches: if *S*. *canadensis* has evolved in response to differing climate along elevation gradients in Switzerland, we would expect survival, growth, and/or flowering success to be higher in planting sites that are climatically similar to the source location of a given clone or population. However, in a reciprocal transplant experiment higher performance at home vs. away sites could result from any number of factors (temperature, precipitation, pathogens, soil type), and a very large number of planting sites would be needed to determine how rapidly performance changes with environmental distance. Planting multiple source populations in a smaller number of test gardens reduces this burden, while the fact that a given source may differ greatly from a given garden in temperature but only slightly in precipitation (and vice versa for other sources) enable one to better disentangle the factors driving differences in performance.

Because *S*. *canadensis* is considered invasive in Switzerland, and much effort is expended to control it, federal office approval for our project required the following: (1) limiting the size of the common gardens, (2) surrounding them with 1.2 m electric fences to exclude cattle during the growing season, (3) lining the raised beds with weed barrier cloth, (4) covering all inflorescences with net bags to reduce pollination and contain any seeds, and (5) removing inflorescences as soon as they faded (before mature seeds were produced). We established the common gardens in *Gemeinde* Untervaz, Graubuenden (Fig 1). The gardens were positioned at three elevations: (1) well inside the elevation and climate limits of *S*. *canadensis* at 659 m (46.938820° N, 9.538261° E), (2) near the upper range limit at 1253 m (46.927003° N, 9.509478° E), and (3) above the normal range limit at 1680 m (46.942859° N, 9.502362° E). The low-elevation garden had two clumps of wild *Solidago* within 200m (not included as source populations in this study due to 5 individual minimum), while the higher sites had no local populations. While we would have liked to include a very-low-elevation garden, we were unable to do so due to the density of human settlement at the lowest elevations (most of our low-elevation samples were collected from sites such as gravel parking lots and railway edges, rather than large open fields where gardens could be placed) and the difficulty of obtaining permissions from land owners.

**Fig 2:**
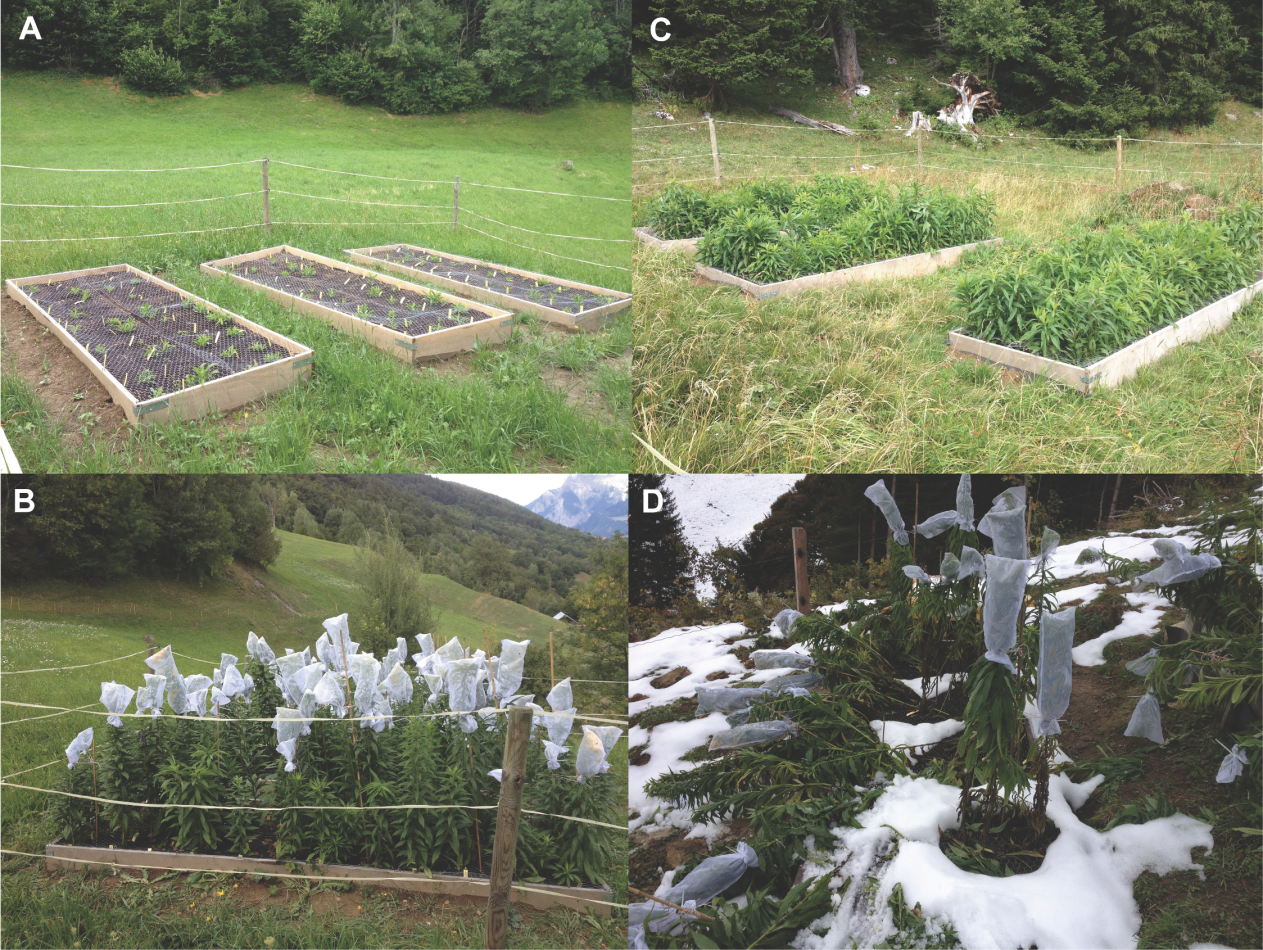
Photos of common gardens. (A) Lowest garden, June 25, 2013. Plants just starting to send up shoots. (B) Lowest garden, September 9, 2013. Inflorescences covered with mesh bags to reduce pollination and prevent seed dispersal. (C) Highest garden, August 26, 2013. Notice that none of the plants have inflorescence buds. (D) Middle elevation garden, October 14, 2013. Stems knocked over by first snowfall.

Each garden contained three 1 x 3 m raised beds to control for edaphic conditions that may vary with elevation and isolate climate effects (Fig 2). The beds were lined with water-permeable weed-barrier cloth, and filled with a 25 cm depth of sand-loam mix. Each was planted with 48 *Solidago* rhizomes, spaced 25 cm apart in two lines of 24 down the length of the bed. There were thus a total of 144 plants per garden. The plants were obtained by clonally propagating rhizome segments from the sampled populations in the greenhouse for 8 months in order to replicate genotypes across elevations. Plants were divided with the goal of planting one replicate per clone in each bed in each garden (3 replicates/garden, 9 total). Not all individuals grew well enough in the greenhouse to be divided into 9 replicates, while others could be divided into 10 or more. Thirteen populations (Fig 1; S1 Table) out of the original 43 examined in the population genetic analyses were selected for inclusion in the common gardens. Inclusion was decided on the basis of whether the population had 4–5 clones with 4–11 replicates each, and representation of the widest possible range of home elevations and population genetic diversity values. Of the 53 clones planted, 25 had the target number of 9 replicates, 3 had 4–5 replicates, 18 had 6–8 replicates, and 7 had 10–11 replicates (S2 Table).

We planted the rhizomes in May of 2013, soon after snowmelt, randomizing the positions of clones within beds. Wire mesh was tacked over the beds to prevent disturbance from digging animals. Every week, we measured the maximum height, shoot number, and the presence and state of flowers on all plants in both 2013 and 2014. As mentioned above, inflorescences were covered with mesh bags before flowers opened and were clipped as soon as flowers faded to reduce pollination success and prevent seed dispersal. Faded inflorescences are referred to as "mature". The end of the growing season was defined as the point when either aboveground tissues were killed by cold or flowering stems were snapped by wind/snow (Fig 2). We calculated height growth rate during the first 60 days after first bolting (stem height growth) in each year. Plants and beds were removed at the end of the 2014 growing season.

### Statistical analysis of common garden experiment

All analyses were conducted in R (version 3.2.1). We first used simple single-variable regression analyses to identify which predictor variables were associated with higher or lower survival, growth rate, and flowering. Predictor variables included garden site; clone identity; population of origin; difference between home site and planting site in each of the environmental variables; overall environmental distance between home and planting site (based on PCA); and number of rhizome buds (which can grow into new ramets) or rhizome mass at time of planting. We then constructed a set of hierarchical Bayesian models for each response variable in each year and used Predictive Loss [72] to determine which combination of predictor variables best explained the results. Unlike AIC or BIC, Predictive Loss (D) does not require one to specify model dimension (number of parameters), which is necessary when dealing with a hierarchical structure such as we used for the clone effects (which are drawn from a distribution which itself has parameters) [73]. Unlike Bayes factor, it is not biased toward the simpler of a set of nested models. The hierarchical Bayesian approach enables us to borrow strength across individuals to better estimate all parameters–for instance, all individuals of clone A (regardless of plot) contribute to the estimation of the clone effect, while all individuals in plot X (regardless of genotype) contribute to the estimation of the plot effect [74]. Due to potential confusion with the genetic differentiation measure D, we will refer to the predictive loss measure as D_m_ because it is dependent on model specification.

For all responses (survival, growth, and flowering) we used a generalized linear model (GLM) framework. In all the models described below, *X* is the design matrix of predictor variables, *ρ* a vector of parameters for fixed (*β*) and random (*α*) effects associated with the predictor variables, and *ε* a Gaussian error term with mean = 0 and variance = *σ*^*2*^. The fixed effects include climate differences, site, etc., while clone identity is treated as a random effect, which we assume to be drawn from a normal distribution such that: $\alpha ∼N(C,S^{2})$. For models with only fixed effects:

$$P(\beta ,\sigma^{2}|Y)\propto P(Y|\beta ,\sigma^{2})P(\beta )P(\sigma^{2})$$

For models with random clone effect:

$$P(\beta ,{\alpha ,\sigma }^{2},C,S^{2}|Y)\propto P(Y|\beta ,\alpha ,\sigma^{2})P(\alpha |C,S^{2})P(\beta )P(\sigma^{2})P(C)P(S^{2})$$

We analyzed years separately because some year effects were likely due to both differences in weather and to plant age, but the two cannot be disentangled with this dataset.

For survival, in which 1 denotes survival and 0 mortality, we used Bernoulli process with a logit link to a linear equation with random effects for clone and fixed effects for other variables.

Inotherwords:Surv∼Bern(θ)logit(θ)=Xρ+ε

For height growth rate, we used a simple linear mixed model:

$$HGR∼N(\mu ,\sigma^{2})\;\mu =X\rho$$

For inflorescence production (referred to below as "flowers" for brevity), we again use a Poisson distribution of flower counts with a log link to a linear equation with random effects for clone and fixed effects for other variables. So in this case:

Flowers∼Poisson(λ)ln⁡(λ)=Xρ+ε

Only living individuals were included in growth and flowering models.

For all three analyses, we tested a wide range of variable combinations. Where *m* is the model variant in question:

$$D_{m}=\overset{n}{\underset{i=1}{\sum }}{\left(E[y_{i}|\vec{y}]-y_{i}\right)}^{2}+\overset{n}{\underset{i=1}{\sum }}var[y_{i}|\vec{y}]$$

D_m_ can be easily calculated from the output of a Gibbs sampler. As with other model selection metrics, one aims to minimize D_m_. We tested all three analyses with simulated data to ensure that “true” parameters could be recovered and that D_m_ would accurately detect over-fitting. Information about the Gibbs Sampler and justification for the priors is given in S3 File. D_m_ values for all models tested are given in S6 File.

## Results

### Population genetic structure

**Fig 3:**
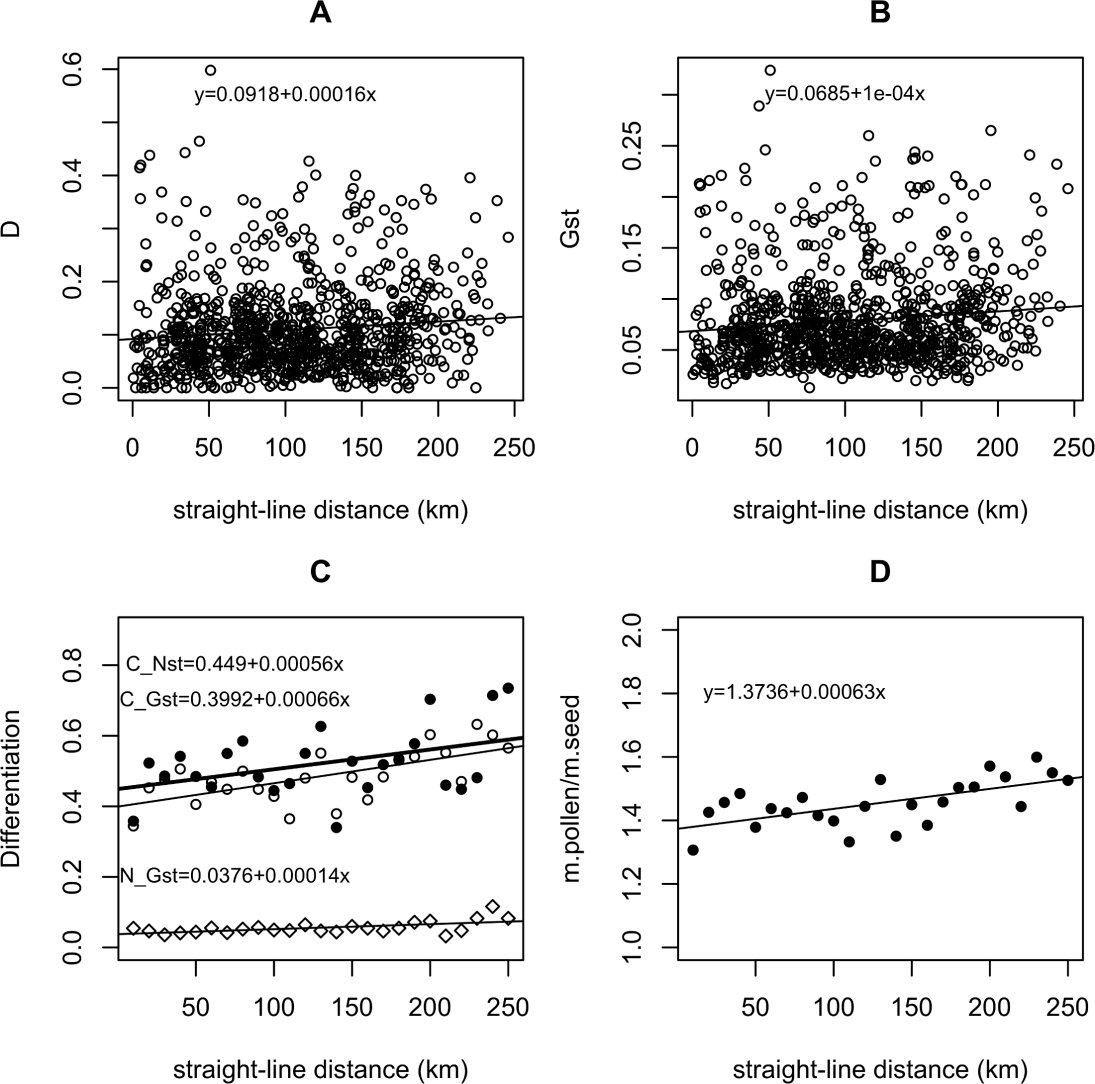
Differentiation measures and estimated ratio of pollen to seed movement versus distance. (A)Pairwise D for nuclear microsatellite markers versus straight-line distance between populations. (B) Pairwise microsatellite G_ST_ versus straight-line distance. (C) Comparison of chloroplast haplotype G_ST_ (open circles, thin line), chloroplast haplotype N_ST_ (black circles, thick line), and average nuclear microsatellite G_ST_ (diamonds, thin line), by 20 m distance class. (D) Estimated ratio of pollen movement (m.pollen) to seed movement (m.seed) by 20 m distance class, based on chloroplast haplotype G_ST_ versus average nuclear microsatellite G_ST._ Linear regression results shown—all are statistically significant.

We found a weak relationship between genetic and geographic distance for both SSR and chloroplast markers consistent with a high degree of gene flow. The 7 SSR loci exhibited a total of 4 to 17 alleles each over all populations (S1 File). The average number of SSR alleles per locus (over all loci) varied between populations from 2.14–3.85. SSR markers exhibited weak but statistically significant differentiation between populations, and a modest increase in differentiation with distance (Fig 3A and 3B; Table 1). Overall G_ST_ was 0.102, and overall D was 0.111. Estimated values of D were generally larger than G_ST_ for most populations, as expected given this measure’s greater sensitivity for loci with more than two alleles. Most pairwise differentiation estimates were less than 0.1 for both D and G_ST_, even at a distance of 250 km. The highest values of pairwise differentiation come from just 3 populations, HH1, HL2, and MM2, which had an average pairwise nuclear G_ST_ >0.14 and D > 0.2 (Figure A in S4 File). These populations were from relatively high elevation areas with low human population density, and so may be unusually isolated (Figure A in S4 File). When these populations were removed from the analysis, maximum pairwise population differentiation at nuclear markers declined (from 0.324 to 0.163 for G_ST_ and 0.598 to 0.2936 for D). The same pattern of a weak increase in genetic differentiation with distance held whether we analyzed all individuals, a subset of 5 individuals per population (the minimum size of populations sampled), or a subset of 11 individuals per population for the 32 populations with at least this many individuals (results not shown). Mantel correlations of differentiation with distance increased with the removal of these populations (Table 1), though the correlation was always weak (<0.21). For all analyses, Mantel correlations were stronger for distance by road than for straight-line distance (Table 1; Figures C and D in S4 File). The MMRR analysis of IBE versus IBD revealed no significant IBE for any tested environmental distance paired with either geographic distance measure (as expected for neutral markers), but IBD was almost always statistically significant (Tables B and C in S4 File). This indicates that while movement of pollen and seed is likely modestly limited by distance, there is no evidence of biased dispersal by environment or of selection limiting gene flow at neutral markers.

**Table 1:** Mantel correlations based on microsatellite markers both with all populations and without the three populations that were unusually highly differentiated from all others (likely due to recent founder effects).

**All populations**				
	**Gst vs. straight-line distance**	**Gst vs. road distance**	**D vs. straight-line distance**	**D vs. road distance**
**Mantel correlation**	0.117	0.126	0.109	0.1232
**P**	0.0316	0.0209	0.0385	0.0239
**Minus populations HH1, HL2, & MM3**				
	**Gst vs. straight-line distance**	**Gst vs. road distance**	**D vs. straight-line distance**	**D vs. road distance**
**Mantel correlation**	0.171	0.204	0.170	0.207
**P**	0.0014	0.0001	0.0008	0.0003

When genetic differentiation is examined by distance class, we can see that differentiation increased quite gradually with distance (Fig 3). Mantel correlograms for genetic differentiation vs. distance exhibited significant correlations at short distances (<30 km) when the three most differentiated populations are removed (Figure D in S4 File), but no correlation at any distance class when all populations are included (Figure C in S4 File). The overall Mantel correlation was weak and usually disappeared with the reduced sample size per distance bin. The Rousset test [61] showed that Gst/(1-Gst) increases slowly with the natural log of distance, with the relationship being somewhat stronger for distance-by-road and when the most differentiated populations were removed (Figure E in S4 File). However, while a linear model could be fit to the relationship between Gst/(1-Gst) and distance (p-values for the distance effect <0.05), the relationship was not very strong for either straight-line or road distance, with an R^2^ of around 0.005 when all populations are included and an R^2^ of around 0.03 when the three most differentiated populations are removed. This casts some doubt on whether the stepping-stone model of IBD applies here [59].

**Fig 4:**
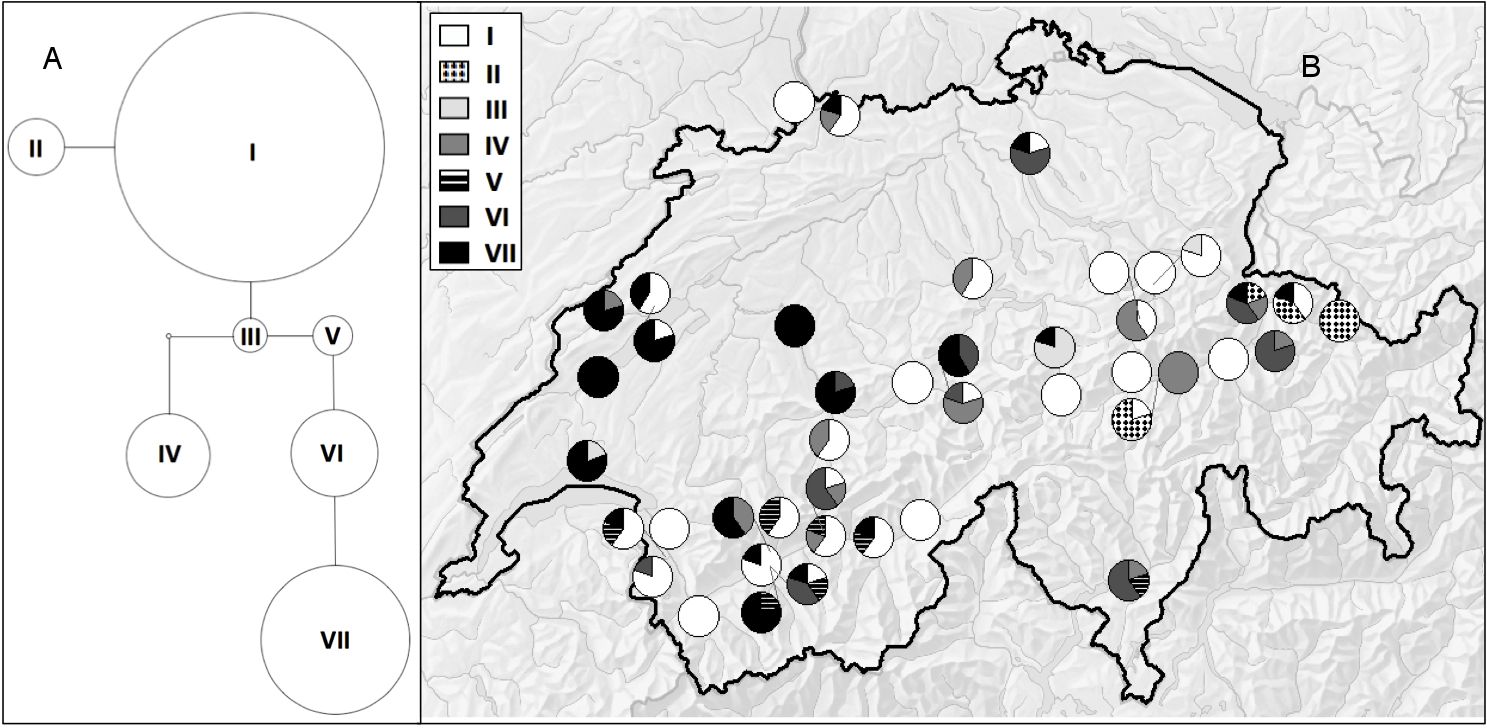
Chloroplast haplotypes. (A) Haplotype network based on statistical parsimony. Circle size corresponds to the number of genotyped individuals with that particular haplotype, while nodes between circles represent the number of changes separating two haplotypes. (B) Distribution of haplotypes across sampled populations in Switzerland. Each pie chart corresponds to one of the sampled populations.

For the chloroplast markers, four phylogenetically informative (PI) polymorphisms were identified (1 nucleotide substitution, 3 insertion/deletion mutations), comprising seven haplotypes (Fig 4A). Haplotype I was the most common, occurring in most populations. Haplotype VII was also widespread, but somewhat more common in the west, whereas haplotypes II and V are confined to populations in the east and south, respectively (Fig 4B). N_ST_ was not significantly larger than G_ST,_ indicating no phylogenetic structure in chloroplast diversity–populations were not more likely to contain closely related haplotypes than distantly related haplotypes. However, both were significantly greater than zero (Table 2), indicating that there is spatial structure in the distribution of haplotypes. Both N_ST_ and G_ST_ increased significantly with distance (Fig 1C; Table A in S4 File). However, for both nuclear and chloroplast markers the rate of increase in the differentiation measure with distance was very gradual, with mean G_ST_ at 240 km being just 1.58x G_ST_ at 20 km for both the chloroplast and nuclear markers. Based on these measures, we calculated that the ratio of pollen to seed movement is 1.41 overall, and increasing from 1.34 at 20 km to 1.57 at 240 km (Fig 1D).

**Table 2:** Average within-population gene diversity (hS, vS) and total gene diversity (hT, vT) used to calculate genetic differentiation (G_ST_, N_ST_) between Swiss populations of *Solidago canadensis* for unordered (hS, hT, G_ST_) and ordered (vS, vT, N_ST_) chloroplast haplotypes.

	hS	hT	G_ST_	vS	vT	N_ST_
Observed	0.38	0.736	0.484	0.348	0.736	0.527
SE	0.046	0.036	0.057	0.047	0.035	0.064

### Adaptation to climate: Survival, growth, and flowering

If populations are adapted to the climatic conditions of their home sites, we would expect to observe higher survival, growth, and/or flowering success at shorter climatic transfer distances. It should be noted that, because of the locations of the common gardens we were able to obtain, most individuals were planted at sites that were colder than their home site in terms of degree days and July temperature. As would be expected, plants started growing earlier and grew faster under warmer conditions (Figures A and B in S5 File). Plants at lower elevations grew taller (Figure A in S5 File), while plants at higher elevations produced more shoots (Figure B in S5 File). Because each shoot can produce one inflorescence, plants with more shoots tended to produce more buds and more mature inflorescences if the growing season was long enough (Figure B in S5 File). Plants produced up to 8 shoots in 2013, and up to 32 in 2014. However, because shoots <60 cm in height did not form inflorescence buds, and because plants at higher elevations gained height more slowly (Fig 2B vs. 2C) and experienced a shorter growing season, this relationship between shoot number and reproductive success was only realized at lower elevations (Figure B in S5 File). Only plants at the lowest site finished flowering before snow or wind broke stems in 2013 (Fig 2D). Most plants at the lowest site and some at the middle site finished flowering in the longer growing season of 2014 (Figure A and Table A in S5 File).

Unexpectedly, survival was higher at higher elevations, ranging from 72% at the 659 m site to 95% at the 1698 m site (Table A in S5 File). The best-fit model for survival (Model S15 in S8 File) included transfer distance for all environmental variables, as well as initial size and clone identity. However, nine other model variants had roughly equivalent fits, with Dm values within the amount that Dm was observed to vary for a single model between runs (S7 File). Of the 10 best models, all included strong initial size effects, with positive effects of rhizome bud number and initial mass. All included site and/or environmental distance effects. If site effects were included, they were more positive for higher elevation sites. Of the environmental distance effects, the strongest and most consistent effects were a positive effect of increased frost index and a negative effect of higher precipitation or annual radiation. The positive impacts of cool conditions and high elevations may reflect higher rhizome establishment success in such areas. The two best models out of the 10 also include clone effects.

**Fig 5:**
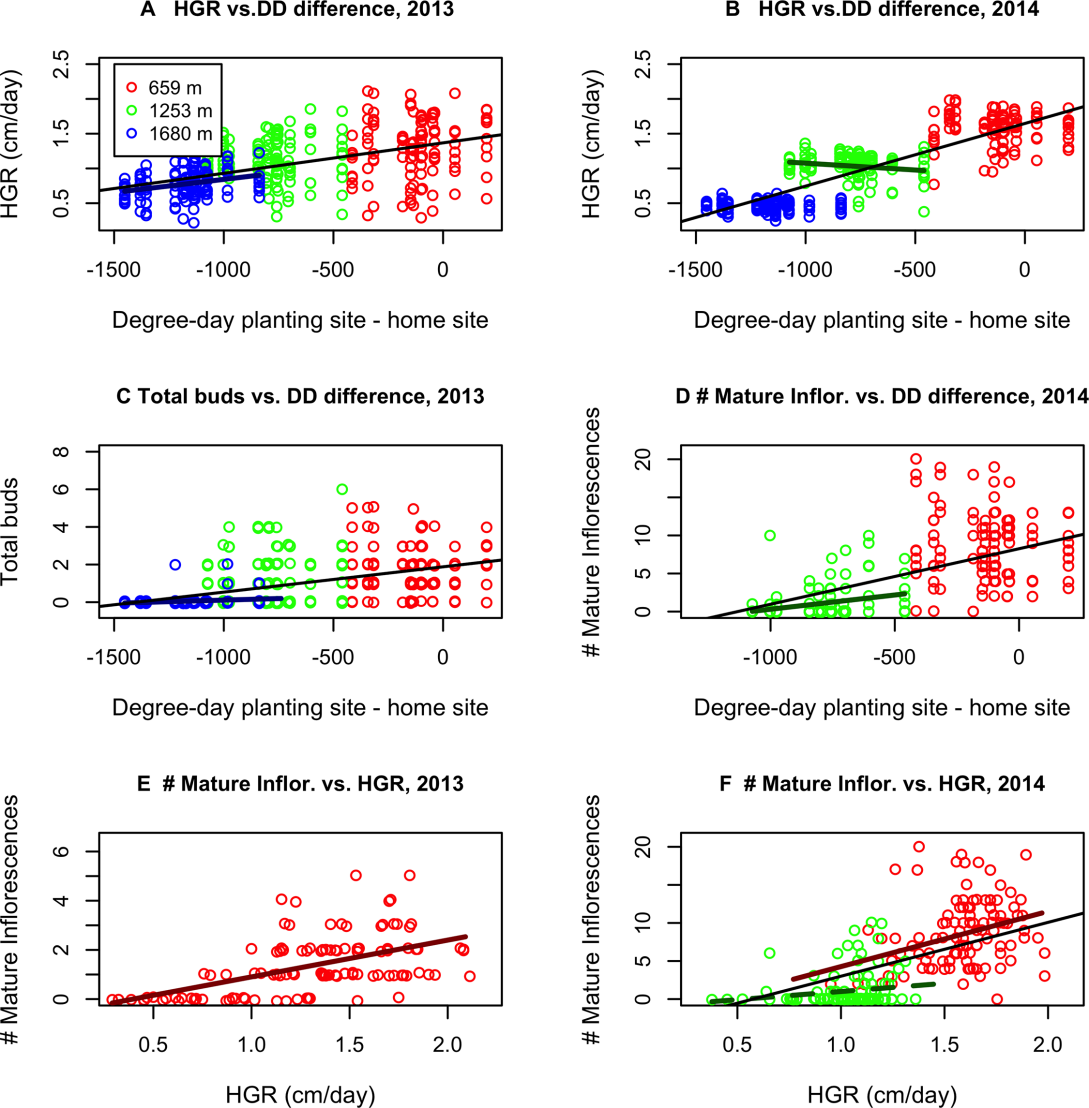
Performance of plants when planted at 3 common gardens along an elevation transect. (A) Height growth rate (HGR) versus difference in average degree-days (DD) between planting site and home site in 2013. (B) HGR vs. difference in DD between planting site and home site in 2014. (C) Total inflorescence buds vs. DD difference in 2013. (D) Number of mature inflorescences vs. DD difference in 2014. E & F—Number of mature flowers vs. HGR in 2013 & 2014. Inflorescences matured (flowers senesced) before end of growing season only at lowest site in 2013 and low and middle sites in 2014. Thick colored lines indicate within-site relationships. Thin black lines indicate across-site relationships. Solid lines indicate statistically significant relationships. Dashed lines indicate marginally significant relationship (p<0.1).

Across all garden elevations, plants grew faster when garden climate was more similar to the climate of their origin, particularly in terms of degree days (Fig 5A and 5B), but also in terms of overall climate distance, July temperature, and, for plants transferred to frostier sites, growing season frost index (Figure C in S5 File). Plants from colder populations grew significantly faster at the highest site in 2013 than plants from warmer populations (Fig 5A; Figure C in S5 File). Average clone HGR at the highest site was marginally positively associated (p<0.1) with home elevation in both years (FigureD in S5 File). However, plants from warmer populations grew faster at the middle site in 2013 (Fig 3B).

The overall best-fit models for HGR included site identity, clone identity, and initial size in 2013 (Model G13 in S6 File) and these factors plus the difference in precipitation distance in 2014 (Model G16b in S6 File). However, six other model variants had roughly equivalent fits for 2013 and 10 others had roughly equivalent fits for 2014 (S8 File). Of these models, all included clone effects, and most included site effects, with growth being slower at high elevations. For 2013 growth, all of the best-fit models include initial mass (which has a strong positive effect) and most include initial number of rhizome buds (which has a weak negative effect). For 2014 growth, the initial size effects are weaker, with 7 of the top 8 models including a modest positive effect of initial mass, but only one showing an effect of initial rhizome buds. Environmental distance effects were relatively weak in these models that included site effects, consistent with the weaker relationship between growth and degree-day distance within sites relative to across all sites shown in Fig 5, but 5 of 7 of the best-fit models for 2013 and 7 of 10 for 2014 included at least one environmental distance effect.

Inflorescences formed earlier on plants with high height growth rate (HGR) in all sites and years, and the earlier the first buds formed, the more likely plants were to complete flowering by the end of the season (Figure E in S5 File). As a result, plants both within and between sites that exhibited early fast growth tended to produce more total inflorescence buds ("total flowers") and, if the growing season was long enough, more mature inflorescences (Fig 5E and 5F).

**Fig 6:**
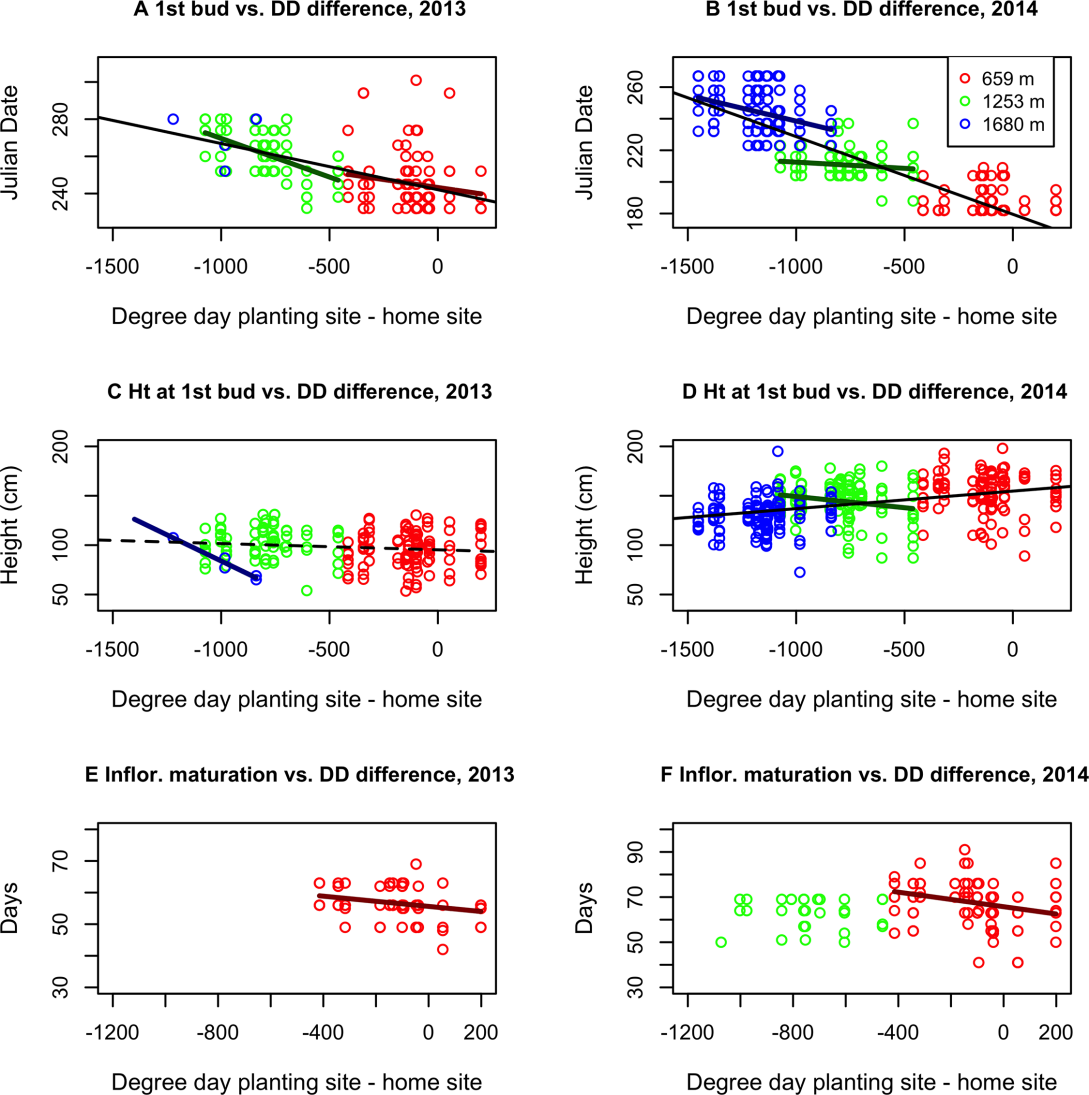
Additional performance measures relative to difference in degree days between planting site and home site. (A) Julian date at which first inflorescence bud recorded, 2013. (B) Julian date of first inflorescence bud, 2014. (C) Plant height at date of first inflorescence bud, 2013. Stems stop growing once apical meristem transforms. (D) Plant height at date of first flower bud, 2014. (E) Time between first bud formation and first inflorescence maturation, 2013. (F) Time between first bud formation and first maturation, 2014. Thick colored lines indicate within-site relationships. Thin black lines indicate across-site relationships. Solid lines indicate statistically significant relationships. Dashed lines indicate marginally significant relationship (p<0.1).

The effects of climate transfer distance on flowering were more pronounced in colder sites. At the site above the current elevation range edge, though no plants were able to complete flowering before the end of the growing season, plants from colder home sites grew faster in 2013 (Fig 5A; Figure C in S5 File) and were the only ones to produce buds (Fig 5C). Among those that that did produce inflorescence buds, those from colder sites produced them at shorter stem heights (Fig 6C). In 2014, plants from colder sites produced buds earlier in the season at the highest site (Fig 6B). At the elevation range edge site, plants from colder home sites produced buds earlier in 2013 (Fig 6A) and produced more mature inflorescences in 2014 (Fig 5D) despite growing slower (Fig 5B; Figure C in S5 File). This may be because they produced buds not only earlier (Fig 6B) but at lower heights (Fig 6D) in this year. At the site within the current range, plants from colder home sites produced inflorescence buds earlier in 2013 and their flowers matured faster in both years (Fig 6A, 6E and 6F), suggesting a genetic predisposition to faster development. However, there were no clear differences in flower production by transfer distance at this site.

Total inflorescence bud numbers in both years were best explained by a model including planting site, all environmental distances, initial size, HGR, and clone identity (Model F22 in S6 File and S9 File). However, model F21 (same minus site effects) had a roughly equivalent fit for both years, while model F18 (same minus initial size effects) had a roughly equivalent fit for 2013 (S9 File). These models agree that in both years higher HGR is strongly associated and larger initial size is weakly associated with higher bud number, while higher precipitation relative to the home site is negatively associated with total inflorescence buds. Other environmental effects differed between years, and show evidence of some tradeoffs in estimated sign and magnitude due to correlations between variables. Mature inflorescences in 2013 were also best explained by model F22, though four other models had roughly equivalent fits (S9 File). All included site and HGR, while 3 included environmental distance effects, 2 included clone effects, and one included population effects. In 2014, a model that included only site and HGR (Model F7 in in S6 File) best fit mature inflorescences, though model F22 was a roughly equivalent fit (S9 File). Across both years, these models agree that higher HGR and higher initial mass is strongly associated with mature flowers, while higher elevation sites are associated with fewer mature inflorescences.

While average clone growth rate was positively correlated across sites (Figure F in S5 File) and years, indicating that certain clones tend to be faster- or slower-growing regardless of environment, bud production was positively correlated between the middle and upper sites in both years, marginally correlated between the lower and middle sites in the 2014, and not correlated between the lower and upper sites (Figure G in S5 File). Thus, a relatively consistent ranking of clone growth rates across sites did not result in a consistent ranking of flowering success—different clones had higher or lower flowering success at different elevations. This result is consistent with clones differing in their sensitivity to flowering cues such as photoperiod, and suggests that shorter growing seasons may independently select for faster growth and different flowering-cue sensitivities. Finally, clones within populations exhibited substantial variation in growth rate and flowering success (Figure H in S5 File). This suggests that there is substantial genetic variation present in these traits upon which natural selection can act.

Overall, plants from colder home sites tend to exhibit a number of traits consistent with adaptation to their location along the climatic gradient. These include, under cold conditions, more rapid growth, inflorescence bud formation at shorter heights, earlier bud formation, and more rapid flower maturation. This was not sufficient to enable flower maturation beyond the current upper range boundary in either of our monitoring years, but did enable higher flower maturation at the range-edge site in 2014. Clones that tend to produce more buds at the upper range-edge site also produced more buds at the beyond-range-edge site and vice versa, but high inflorescence bud production at these sites did not predict production at the within-range site.

## Discussion

We found that gene flow in populations of invasive *S*. *canadensis* is likely fairly extensive, but not sufficient to prevent population differentiation and adaptation along elevation climate gradients. Specifically, we found that population genetic differentiation increased only slowly with distance. Nonetheless, plants grown in common-gardens exhibited some adaptation to climate, with clones from colder home sites performing better at or beyond the species’ high elevation range limit compared to clones from warmer home sites. This may indicate a potential for adaptation to facilitate range expansion, especially as climate warms.

### Introduction history and gene flow

We found evidence of weak but statistically significant overall population differentiation and weakly increasing differentiation with geographic distance in the nuclear SSR markers and chloroplast haplotypes. The interpretation of genetic differentiation statistics can be complex, because these values are highly dependent on the test’s assumptions about the number of alleles per locus [57], as well as the mutation rate of the loci and the migration rate between populations. For mutation rates around 10^−3^ per generation, the values of D and G_ST_ observed for Swiss *S*. *canadensis* populations are consistent with 0.01–1 migrants/generation between most populations [75,76]. A gene flow rate of ~1 migrant/generation is generally considered sufficient to prevent divergence due to drift [77].

Patches of *Solidago* exist in a shifting mosaic connected by dispersal events, with disturbance being required for seedling establishment and patch extinctions caused by further disturbance or ecological succession to woody vegetation. The stronger correlation of genetic differentiation with road distance than with straight-line distance and with geographic distance rather than environmental distance is consistent with this life history. Vegetation-removing disturbances are common along roads due to construction and maintenance activity. Seed dispersal can also be facilitated by the "wind" produced by passing vehicles or the mud stuck to tires. Roads have been found to facilitate the spread of a variety of plants, particularly introduced species [78,79]. The Rousset test indicated that the stepping-stone model of IBD may not be the best explanation for the relationship between genetic and geographic distance. This could arise from the role of human transport in the spread of *Solidago*. While much human transport would also be local (eg. gardeners sharing plants), longer distance shipments between nurseries likely took place with some frequency.

There was no phylogeographic structure to the chloroplast haplotype data; haplotypes separated by few mutations were not closer to each other in space than would be expected by chance. This suggests that several maternal lines (existing haplotypes) were introduced to Switzerland, rather than a scenario where mutations accumulated during spread resulting in the clumping of related haplotypes. Comparison of G_ST_ for chloroplast and nuclear markers and the very gradual increase in differentiation with distance suggests that the movement of pollen is somewhat higher than the movement of seeds/vegetative units. Plants with heavier seeds tend to have a much higher ratio of pollen to seed movement [77], but *Solidago* has both small, wind-dispersed seeds (20–25% can travel > 100 m under the right conditions [52]), a variety of insect pollinators, some capable of travelling long distances (e.g. 0.1–100 km for bees [51]), and the aid of human transport of plants or seeds. It should be noted that chloroplast sequence mutation rates are lower than SSR repeat-number mutation rates, which might make the ratio m_p_: m_s_ to appear smaller than it is, although the tendency for this ratio to be >1 for most plants is robust to the type of nuclear marker used [80].

### Adaptation to climate

*S*. *canadensis* populations in Switzerland show signs of adaptation to climate differences along elevation gradients in ways that are relevant for population expansion into colder environments. In colder sites and years, individuals from colder and/or higher home sites tended to exhibit faster growth, earlier inflorescence bud formation, and/or more total buds. Fast growth and early flowering are vital in cold environments, as the main constraint on fitness is the ability to produce seed before winter. In the first year, which had a short growing season, only a few “high elevation” individuals produced buds at the highest (1680 m) site. However, no plants were able to complete flowering at the highest elevation site in either year. Weber and Schmid [34] found that European populations of *S*. *altissima [S*. *canadensis]* and *S*. *gigantea* from higher latitudes were smaller overall but grew faster and reached flowering sooner than southern populations, paralleling our results along elevation climate gradients.

Surprisingly, however, survival was positively correlated with elevation and with transfer to cooler conditions. It is not clear why this is, but we hypothesize that cool, moist conditions provided better conditions for early root establishment and survival of rhizome transplants, as virtually all mortality occurred in the first few months of the first year. We were also surprised to find that freezing and snow did not substantially damage the leaves of *S*. *canadensis*. Instead, winter wind and snow eventually ended the growing season by breaking shoots, preventing further flower development.

Potential for further adaptation to local climate is evident in the strong clone effects. Despite low genetic diversity within and across populations as measured by SSR markers (1–7 alleles/locus per population (Table C in S1 File); see [41] for direct comparison between European and North American populations), there was pronounced variation between clones in performance. Neutral genetic markers such as SSRs used to assess bottlenecks and population connectivity do not necessarily reflect the diversity of or differentiation in ecologically important functional loci [81,82]. The presence of seven chloroplast haplotypes in Switzerland that do not exhibit phylogenetic spatial structure indicates that at least this number of lineages or cultivars were introduced to Switzerland from elsewhere in Europe. These lineages may have brought with them variation at functional loci.

Survival, growth, and flowering data suggest that the elevation limits of *S*. *canadensis* in Europe are likely set by limits of growing season length on reproduction rather than by the effects of cold on survival. If propagules reach high elevations they can survive, but if the growing season is short this will limit population spread by both vegetative and sexual reproduction. In natural populations, a higher rate of population extinction has been detected near *Solidago's* upper range limit [50]. This could be due to factors that were eliminated in this experiment, such as grazing pressure or competition from other species. The greater potential of individuals from high elevation to grow and flower at or beyond the range boundary suggests that adaptation to climate along elevation gradients likely reduces the decline in fitness that might otherwise be observed as one approaches the range edge. However, the stability of the range edge over at least a decade suggests that there could be a species-level limit to population adaptation to a shorter growing season [40,50].

It is possible that some of the differences between clones or populations could be due to maternal or epigenetic effects. Although growing the clones from a small size in a common environment for eight months prior to planting, and extending the experiment to a second year in the field, would likely reduce clonal difference due to such effects, more persistent changes, such as those arising from heritable methylation, may not have been eliminated[83,84]. However, if maternal or epigenetic effects do persist for two or more years, the consequences of these effects would be similar to genetic differences: increasing the probability of reproductive success at or near the range edge for propagules coming from colder environments.

### Implications for the role of adaptation and gene flow in species invasion

Our finding of growing season length controls over the altitudinal range boundary of *S*. *canadensis* is consistent with other studies of invasive plants. The annual cocklebur, *Xanthium strumarium*, could establish beyond its current range edge if photoperiod cues were manipulated to allow earlier flowering, which suggests that evolution in response to photoperiod might enable range expansion [85]. The evolution of earlier flowering in northern populations of *Lythrum salicaria* in North America seems to have had such an effect on the range [23]. Such constraints may be particularly strong on fall-flowering species and those in which reproduction is strongly related to size. Species such as *S*. *canadensis*, where survival is high at or beyond the range edge, but reproduction is limited by the growing season length, may be able to expand their ranges rapidly if climate change lengthens the growing season, especially if populations near the current elevation or latitudinal limit have already evolved faster growth.

Our results show that at least some degree of adaptation to climate in an introduced species can occur even in the face of fairly extensive gene flow. Because *Solidago canadensis* has relatively low genetic diversity in Europe, and good dispersal abilities, this potential for adaptation even across steep environmental gradients likely holds for many other invasive species with similar or more restricted dispersal abilities. This is especially true for those that have experienced multiple introductions that boost their genetic diversity. We therefore conclude that the potential for partial adaptation to cold climates by range edge populations should be more widely considered when projecting climate change impacts on invasive species range expansions.

## Supporting information

S1 Table Populations sampled for population genetic analysis; populations planted in common garden shown in bold. (DOCX)

S2 Table Number of clones per population and replicates per clone planted in common gardens. (DOCX)

S1 File Microsatellite procedures and results by locus. (DOCX)

S2 File Chloroplast sequence procedures. (DOCX)

S3 File Bayesian model setup. (DOCX)

S4 File Additional population genetic structure results. (DOCX)

S5 File Growth and flower production across common gardens. (DOCX)

S6 File All Bayesian models tested, with predictive loss values. (DOCX)

S7 File Best fitting survival model parameters. (DOCX)

S8 File Best fitting height growth model parameters. (DOCX)

S9 File Best fitting flowering model parameters. (DOCX)
