# Supplementary material for: Population genetics and adaptation to climate along elevation gradients in invasive Solidago canadensis
Source: PLoS One. 2017 Sep 28;12(9):e0185539. doi: 10.1371/journal.pone.0185539 (PMC5619793; doi:10.1371/journal.pone.0185539)
Supplement: S1 Table — (DOCX) [file pone.0185539.s001.docx]

**S1 Table: Populations sampled for population genetic analysis; populations planted in common garden shown in bold.**

| Pop | Elev. (m) | # Samples | Lat (N)/Long (E) | Closest Town (Canton) |
| --- | --- | --- | --- | --- |
| HH1 | 814 | 8 | 46.91425°/ 9.77062° | Kublis (GR) |
| **HL1** | **816** | 13 | 46.22196°/ 7.39749° | Sutor (VS) |
| HL2 | 923 | 16 | 46.26786°/ 7.3992° | Blignou (VS) |
| HL3 | 933 | 5 | 46.32869°/ 7.62942° | Tschingere (VS) |
| HL4 | 829 | 17 | 46.96529°/ 6.77806° | Fretereules (NE) |
| **LH1** | **450** | 18 | 47.4188°/ 8.54444° | Zurich (ZH) |
| **LH2** | **452** | 5 | 46.25408°/ 6.94515° | Monthey (VS) |
| LH3 | 599 | 12 | 46.29993°/ 7.53666° | Sierre (VS) |
| LH4 | 614 | 16 | 46.84828°/ 9.5343° | Chur (GR) |
| LH5 | 450 | 15 | 46.98012°/ 6.89247° | Neuchatel (NE) |
| **LH6** | **449** | 16 | 46.77295°/ 6.64967° | Yverdon-Les-Bains (VD) |
| LH7 | 454 | 11 | 46.52792°/ 6.60413° | Laussane (VD) |
| LH8 | 454 | 19 | 47.05884°/ 8.28629° | Luzern (LU) |
| **LH9** | **569** | 6 | 46.9235°/ 7.50588° | Muri-Bei-Bern (BE) |
| LH10 | 257 | 19 | 47.5726°/ 7.60894° | Basel (BS) |
| **LL1** | **475** | 18 | 47.03169°/ 9.07843° | Ennenda (GL) |
| **LL2** | **600** | 18 | 46.22638°/ 7.39814° | La Crettaz (VS) |
| LL4 | 512 | 7 | 46.77532°/ 8.66803° | Amsteg (UR) |
| LL5 | 578 | 18 | 46.75757°/8.01897° | Brienz (BE) |
| **LM2** | **543** | 16 | 46.99625°/ 9.08463° | Schwanden (GL) |
| LM3 | 433 | 18 | 46.2513°/ 7.02232° | Bex (VS) |
| LM5 | 503 | 19 | 46.22837°/ 7.36053° | Sion (VS) |
| **LM6** | **544** | 16 | 46.96924°/ 9.57623° | Landquart (GR) |
| LM7 | 191 | 18 | 46.17386°/ 8.94648° | Gudo (TI) |
| LM8 | 462 | 16 | 46.85442°/ 8.64098° | Schattdorf (UR) |
| LM9 | 592 | 16 | 46.73029°/ 8.18113° | Meiringen (BE) |
| **LM10** | **276** | 7 | 47.53437°/ 7.7093° | Pratteln (BL) |
| **MH1** | **667** | 16 | 46.30697°/ 7.96317° | Brig (VS) |
| MH2 | 640 | 11 | 46.3122°/ 7.6383° | Leuk (VS) |
| MH3 | 640 | 11 | 46.68463°/ 7.68241° | Spiez (BE) |
| ML1 | 630 | 9 | 46.93524°/ 9.01494° | Ruti (GL) |
| ML2 | 536 | 17 | 46.33298°/ 6.9638° | Vers-Morey (VS) |
| ML3 | 655 | 16 | 46.08039°/ 7.09124° | Bovernier (VS) |
| ML4 | 766 | 17 | 46.76015°/ 9.09155° | Run (GR) |
| ML5 | 659 | 7 | 46.96787°/ 9.68887° | Schiers (GR) |
| **ML6** | **782** | 6 | 47.01676°/ 6.87278° | Coffrane (NE) |
| **ML7** | **661** | 14 | 46.69593°/ 8.23916° | Innertkirchen (BE) |
| MM1 | 645 | 20 | 46.92752°/ 9.0012° | Linthal (GL) |
| MM2 | 796 | 7 | 46.35865°/ 8.04812° | Morel (VS) |
| MM3 | 744 | 18 | 46.77625°/ 9.145° | Ruen (GR) |
| MM4 | 718 | 7 | 46.77444°/ 9.17839° | Schnaus (GR) |
| MM5 | 660 | 18 | 46.80917°/ 9.39982° | Bonaduz (GR) |
| MM6 | 777 | 13 | 46.59111°/ 7.65482° | Frutigen (BE) |
