# Supplementary material for: Population genetics and adaptation to climate along elevation gradients in invasive Solidago canadensis
Source: PLoS One. 2017 Sep 28;12(9):e0185539. doi: 10.1371/journal.pone.0185539 (PMC5619793; doi:10.1371/journal.pone.0185539)
Supplement: S2 Table — (DOCX) [file pone.0185539.s002.docx]

**S2 Table:** Number of clones per population and replicates per clone planted in common gardens

| Population | Elev. (m) | Clone | # replicates by garden | Total replicates |
| --- | --- | --- | --- | --- |
| **HL1** | **816** | 4 | L -3, M - 2, H - 2 | 7 |
|  |  | 6 | L -2, M - 2, H - 2 | 6 |
|  |  | 10 | L -3, M - 2, H - 2 | 7 |
|  |  | 12 | L -3, M - 3, H - 3 | 9 |
| **ML6** | **782** | 1 | L -4, M - 4, H - 3 | 11 |
|  |  | 3 | L -2, M - 1, H - 1 | 4 |
|  |  | 4 | L -3, M - 3, H - 3 | 9 |
|  |  | 6 | L -3, M - 3, H - 3 | 9 |
| **MH1** | **667** | 3 | L -3, M - 3, H - 3 | 9 |
|  |  | 10 | L -3, M - 3, H - 3 | 9 |
|  |  | 11 | L -3, M - 3, H - 3 | 9 |
|  |  | 15 | L -3, M - 3, H - 3 | 9 |
| **ML7** | **661** | 5 | L -3, M - 3, H - 3 | 9 |
|  |  | 9 | L -2, M - 2, H - 2 | 6 |
|  |  | 10 | L -3, M - 3, H - 3 | 9 |
|  |  | 11 | L -2, M - 2, H - 2 | 6 |
| **LL2** | **600** | 2 | L -3, M - 3, H - 4 | 10 |
|  |  | 8 | L -3, M - 4, H - 3 | 10 |
|  |  | 10 | L -3, M - 3, H - 3 | 9 |
|  |  | 16 | L -3, M - 3, H - 4 | 10 |
| **LH9** | **569** | 1 | L -3, M - 3, H - 3 | 9 |
|  |  | 2 | L -1, M - 2, H - 2 | 5 |
|  |  | 3 | L -3, M - 3, H - 3 | 9 |
|  |  | 4 | L -2, M - 2, H - 2 | 6 |
| **LM6** | **544** | 1 | L -3, M - 3, H - 3 | 9 |
|  |  | 3 | L -2, M - 2, H - 2 | 6 |
|  |  | 10 | L -2, M - 2, H - 2 | 6 |
|  |  | 11 | L -2, M - 2, H - 2 | 6 |
| **LM2** | **543** | 6 | L -2, M - 3, H - 3 | 8 |
|  |  | 10 | L -2, M - 2, H - 2 | 6 |
|  |  | 11 | L -4, M - 3, H - 3 | 10 |
|  |  | 13 | L -3, M - 3, H - 3 | 9 |
| **LL1** | **475** | 3 | L -3, M - 3, H - 3 | 9 |
|  |  | 7 | L -3, M - 3, H - 3 | 9 |
|  |  | 9 | L -3, M - 3, H - 3 | 9 |
|  |  | 13 | L -2, M - 2, H - 2 | 6 |
| **LH2** | **452** | 1 | L -2, M - 3, H - 2 | 7 |
|  |  | 2 | L -3, M - 3, H - 3 | 9 |
|  |  | 4 | L -3, M - 2, H - 3 | 8 |
|  |  | 5 | L -3, M - 4, H - 3 | 10 |
| **LH1** | **450** | 1 | L -3, M - 3, H - 3 | 9 |
|  |  | 2 | L -3, M - 2, H - 2 | 7 |
|  |  | 7 | L -3, M - 3, H - 3 | 9 |
|  |  | 9 | L -2, M - 2, H - 3 | 7 |
|  |  | 13 | L -2, M - 3, H - 3 | 8 |
| **LH6** | **449** | 2 | L -3, M - 3, H - 3 | 9 |
|  |  | 4 | L -3, M - 3, H - 3 | 9 |
|  |  | 5 | L -4, M - 3, H - 3 | 9 |
|  |  | 9 | L -3, M - 3, H - 3 | 9 |
| **LM10** | **276** | 1 | L -3, M - 3, H - 4 | 10 |
|  |  | 3 | L -3, M - 3, H - 3 | 9 |
|  |  | 6 | L -2, M - 1, H - 2 | 5 |
|  |  | 7 | L -2, M - 3, H - 2 | 7 |
