# Supplementary material for: Population genetics and adaptation to climate along elevation gradients in invasive Solidago canadensis
Source: PLoS One. 2017 Sep 28;12(9):e0185539. doi: 10.1371/journal.pone.0185539 (PMC5619793; doi:10.1371/journal.pone.0185539)
Supplement: S1 File — (DOCX) [file pone.0185539.s003.docx]

**S1 File: Microsatellite procedures and results by locus**

Marker screening:

We initially tested 12 microsatellite markers: SS1B, SS4G, SS19C, SS20E, SS4F, SS24F, and SS19D from Wieczorek & Geber [40] and SC40, SC45, SC23, SC51, and SC54 from Zhao et al. [41]. Although the SC markers were developed for Asian populations of *Solidago canadensis*, most failed to amplify. All the SS markers and SC40 produced clear diploid peaks and were polymorphic in our sampled populations. SS19D was excluded from further analyses because of a high frequency of null alleles that caused it to deviate from Hardy-Weinberg (HW) equilibrium in nearly all populations.

Genotyping:

Microsatellite markers were amplified in either a multiplex or simplex polymerase chain reaction (PCR) using either BIOLINE or KAPA3G DNA polymerase (Table 1). All PCR products were separated and detected on a 3130xl DNA analyzer (Applied Biosystems, ABI) and alleles were scored using Geneious Pro 5.6.6 (Biomatters).

The PCRs under the BIOLINE protocol were carried out in a 10 μl reaction volume and included 1 μl of template DNA (with an average concentration of 7.04 ng μl^-1^), 1X reaction buffer, 1.5 mM MgCl_2_, 0.2 mM of each dNTP, 0.3 μM of each primer (0.2 and 0.6 μM for SS19D and SS24F, respectively) and 0.5 U Taq (BIOTAQ^TM^ DNA polymerase, BIOLINE). PCRs included an initial denaturation period (15 min at 95°C), followed by 30 cycles of annealing (30 seconds at a primer specific annealing temperature, refer to Table X), elongation (30 seconds at 75°C), and denaturation (30 seconds at 95°C). The reactions were concluded with a final annealing step (1 minute at a primer specific annealing temperature) and a final elongation step (30 minutes at 75°C).

For the KAPA3G protocol, 10 μl reactions contained 5 μl of 2X KAPA Plant PCR Buffer (including 1.5mM of MgCl_2_ and a manufacturer’s set concentration of dNTPs), 0.2-0.6 μM of each primer (refer to Table 1), 1U of KAPA3G plant DNA polymerase (KAPABIOSYSTEMS 3.11), and 1 μl of template DNA (with an average concentration of 7.04 ng μl^-1^). Cycling parameters included an initial denaturation period (3 min at 95°C); 30 cycles of denaturation (20 sec at 95°C), annealing (15 sec at a primer specific temperature, refer to Table 1), and extension (30 sec at 72°C); and a final extension period (30 sec at 72°C).

Table A: Microsatellite (SSR) regions and the corresponding fluorochrome, annealing temperature (T_A_), and DNA polymerase used for amplification.

| **SSR Region** | **Fluorochrome** | **T_A_** | **DNA Polymerase** |
| --- | --- | --- | --- |
| SS4G | FAM | 46 | Biotaq |
| SS19C | HEX | 46 | Biotaq |
| SS1B | Atto565 | 58 | Biotaq |
| SS20E | FAM | 50 | Biotaq |
| SS4F | HEX | 50 | Biotaq |
| SS19D | Atto565 | 58 | Kapa 3G |
| SS24F | FAM | 59 | Kapa 3G |
| SC40 | FAM | 55 | Kapa 3G |

Results:

Table B: Alleles per locus, expected minus observed heterozygosity, and deviations from Hardy-Weinberg equilibrium

| Locus | Total # alleles observed | Average (H_E_-H_o_) | Populations deviating from HWE | Monomorphic populations |
| --- | --- | --- | --- | --- |
| SS1B | 8 | -0.033 | 4 | 1 |
| SS4G | 17 | -0.051 | 5 | 0 |
| SS19C | 4 | -0.074 | 2 | 0 |
| SS20E | 5 | 0.052 | 10 | 5 |
| SS4F | 5 | -0.010 | 2 | 2 |
| SS24F | 4 | -0.062 | 4 | 0 |
| SC40 | 5 | 0.091 | 11 | 5 |

H_E_: Expected heterozygosity; H_O_: Observed heterozygosity; HWE: Hardy-Weinberg Equilibrium

Table C: Alleles per locus by population. Populations planted in common gardens shown in bold.

| Pop | Elev. (m) | N | SS1B | SS4G | SS19C | SS20E | SS4F | SS24F | SC40 | Mean |
| --- | --- | --- | --- | --- | --- | --- | --- | --- | --- | --- |
| HH1 | 814 | 8 | 1 | 3 | 4 | 3 | 1 | 1 | 2 | 2.14 |
| **HL1** | **816** | 13 | 5 | 4 | 2 | 3 | 3 | 3 | 4 | 3.43 |
| HL2 | 923 | 16 | 4 | 4 | 2 | 2 | 2 | 2 | 1 | 2.42 |
| HL3 | 933 | 5 | 4 | 3 | 2 | 1 | 4 | 3 | 4 | 3 |
| HL4 | 829 | 17 | 4 | 5 | 3 | 3 | 3 | 3 | 2 | 3.29 |
| **LH1** | **450** | 18 | 6 | 5 | 2 | 2 | 2 | 3 | 3 | 3.29 |
| **LH2** | **452** | 5 | 5 | 4 | 2 | 2 | 2 | 3 | 2 | 2.9 |
| LH3 | 599 | 12 | 5 | 5 | 4 | 3 | 2 | 3 | 4 | 3.71 |
| LH4 | 614 | 16 | 3 | 4 | 2 | 2 | 2 | 3 | 3 | 2.71 |
| LH5 | 450 | 15 | 5 | 6 | 4 | 3 | 3 | 3 | 2 | 3.71 |
| **LH6** | **449** | 16 | 5 | 5 | 2 | 3 | 4 | 4 | 1 | 3.43 |
| LH7 | 454 | 11 | 4 | 5 | 2 | 1 | 3 | 3 | 2 | 2.86 |
| LH8 | 454 | 19 | 5 | 3 | 2 | 2 | 2 | 3 | 3 | 2.86 |
| **LH9** | **569** | 6 | 3 | 3 | 2 | 3 | 2 | 3 | 3 | 2.71 |
| LH10 | 257 | 19 | 5 | 6 | 2 | 1 | 2 | 3 | 3 | 3.14 |
| **LL1** | **475** | 18 | 5 | 4 | 2 | 2 | 2 | 3 | 1 | 2.71 |
| **LL2** | **600** | 18 | 6 | 5 | 3 | 2 | 4 | 3 | 4 | 3.86 |
| LL4 | 512 | 7 | 4 | 3 | 2 | 2 | 2 | 3 | 3 | 2.71 |
| LL5 | 578 | 18 | 5 | 8 | 2 | 2 | 2 | 3 | 3 | 3.57 |
| **LM2** | **543** | 16 | 6 | 5 | 2 | 3 | 3 | 3 | 1 | 3.29 |
| LM3 | 433 | 18 | 5 | 8 | 2 | 2 | 3 | 3 | 3 | 3.71 |
| LM5 | 503 | 19 | 4 | 3 | 3 | 3 | 4 | 3 | 4 | 3.43 |
| **LM6** | **544** | 16 | 5 | 3 | 3 | 2 | 1 | 3 | 2 | 2.71 |
| LM7 | 191 | 18 | 5 | 4 | 2 | 2 | 2 | 3 | 2 | 2.86 |
| LM8 | 462 | 16 | 5 | 7 | 2 | 2 | 2 | 3 | 3 | 3.43 |
| LM9 | 592 | 16 | 4 | 4 | 2 | 2 | 2 | 3 | 3 | 2.86 |
| **LM10** | **276** | 7 | 5 | 7 | 2 | 2 | 2 | 3 | 2 | 3.29 |
| **MH1** | **667** | 16 | 5 | 4 | 2 | 2 | 2 | 3 | 3 | 3 |
| MH2 | 640 | 11 | 4 | 5 | 3 | 2 | 2 | 3 | 2 | 3 |
| MH3 | 640 | 11 | 5 | 4 | 2 | 3 | 2 | 3 | 2 | 3 |
| ML1 | 630 | 9 | 5 | 3 | 2 | 2 | 2 | 2 | 2 | 2.57 |
| ML2 | 536 | 17 | 5 | 4 | 2 | 2 | 2 | 3 | 3 | 3 |
| ML3 | 655 | 16 | 4 | 3 | 2 | 1 | 2 | 3 | 2 | 2.43 |
| ML4 | 766 | 17 | 5 | 3 | 3 | 2 | 2 | 3 | 3 | 3 |
| ML5 | 659 | 7 | 6 | 3 | 2 | 2 | 2 | 3 | 4 | 3.14 |
| **ML6** | **782** | 6 | 3 | 3 | 3 | 2 | 2 | 3 | 3 | 2.71 |
| **ML7** | **661** | 14 | 6 | 5 | 2 | 2 | 2 | 3 | 2 | 3.14 |
| MM1 | 645 | 20 | 4 | 5 | 2 | 2 | 2 | 3 | 2 | 2.86 |
| MM2 | 796 | 7 | 5 | 5 | 2 | 2 | 2 | 3 | 3 | 3.14 |
| MM3 | 744 | 18 | 4 | 3 | 2 | 2 | 2 | 3 | 4 | 2.86 |
| MM4 | 718 | 7 | 3 | 2 | 2 | 3 | 2 | 3 | 1 | 2.29 |
| MM5 | 660 | 18 | 5 | 3 | 2 | 2 | 2 | 3 | 3 | 2.86 |
| MM6 | 777 | 13 | 4 | 5 | 2 | 2 | 2 | 3 | 2 | 2.86 |

**References:**

Wieczorek, A.M., and M.A. Geber. 2002. Microsatellite loci for studies of population differentiation and range expansion in *Solidago sempervirens* L. (Asteraceae). Molecular Ecology Notes 2:554-556.

Zhao, S.Y., S.G. Sun, Y.H. Guo, J.M. Chen, and Q.F. Wang. 2012. Isolation and characterization of polymorphic microsatellite loci from the invasive plant *Solidago canadensis* (Asteraceae). Genetics and Molecular Research 11:421-424.

Geneious version 5.6.6 created by Biomatters. Available from <http://www.geneious.com/>
