# Supplementary material for: Population genetics and adaptation to climate along elevation gradients in invasive Solidago canadensis
Source: PLoS One. 2017 Sep 28;12(9):e0185539. doi: 10.1371/journal.pone.0185539 (PMC5619793; doi:10.1371/journal.pone.0185539)
Supplement: S2 File — (DOCX) [file pone.0185539.s004.docx]

**S2 File: Chloroplast marker procedures**

DNA amplification:

We sequenced four regions of the chloroplast genome found to be variable within the genus *Solidago* (trnH-psbA, rpl36-rps8, trnK-rps16, and trnV-atpE; Kress et al. 2005, Schlaepfer et al. 2008) for up to 5 individuals per population of *Solidago canadensis* (N=224). These regions make up a total of 3,222 base pairs. For primer information and references, refer to Table 1.

Table A: Information on chloroplast gene regions used in this study

| **Locus** | **Primer** | **Sequence (5' - 3')** | **Length** | **T_A_*** |
| --- | --- | --- | --- | --- |
| trnH-psbA | trnH-f | CGCGCATGGTGGATTCACAAATC | 411-590 | 52 |
|  | psbA-r | TGCATGGTTCCTTGGTAACTTC |  |  |
| rpl36-rps8 | rpl36f | CACAAATTTTACGAACGAAG | 492 | 48 |
|  | rps8r | TAATGACAGAYCGAGARGCTCGAC | |  |
| trnK-rps16 | trnK5'r | TACTCTACCRTTGAGTTAGCAAC | 412-803 | 48 |
|  | rps16-4547mod | AAAGGKGCTCAACCTACARGAAC | |  |
| trnV-atpE | trnV5f | GTGTAAACGAGTTGCTCTACCA | 418-1270 | 55 |
|  | atpE-S1022 | CGACATTTGCACATTTAGATGCTAC | |  |

*T_A_: Annealing temperature

Polymerase chain reaction (PCR) for chloroplast DNA amplification was conducted in a 50 μl reaction volume, including 2 μl of template DNA (with an average concentration of 7.04 ng μl^-1^), 5 μl reaction buffer (10X), 1.5 μl MgCl_2_ (50 mM), 0.8 μl of each dNTP (2.5 mM), 1.5 μl of each primer (10μM), and 0.5 μl of Taq polymerase (5U/μl; BIOTAQ^TM^ DNA polymerase, BIOLINE).

Sequencing procedure:

Amplified products were purified with Exonuclease I (Thermo Scientific) and directly sequenced following a standard Sanger sequencing protocol. In a 10 μl sequencing reaction volume, 3 μl of purified PCR product was used, along with 1.6 μl sequencing buffer (5X), 1.6 μl sequencing primer (1 μM), and 0.8 μl Big Dye v3.1 (Applied Biosystems, ABI). Cycle sequencing included an initial denaturation period (3 min at 94°C), followed by 30 cycles of denaturation (30 seconds at 94°C), annealing (30 seconds at a primer specific annealing temperature, refer to Table X), and extension (30 seconds at 72°C), and a final extension period (7 min at 72°C).

The cycle sequencing products were further purified using Sephadex filtration and analysed on a 3130xl DNA analyzer (Applied Biosystems, ABI). Sequences were assembled using Sequencher 5.1 (Gene Codes) and aligned using the MUSCLE algorithm in Geneious Pro 5.6.6 (Biomatters). Alignments were then adjusted manually.

The 4-region alignment had no missing polymorphic data, but a mono-A-string presented alignment difficulties. Such repeat regions have a high mutation rate and potential for homoplasy, so the mono-A-string was removed from the alignment (Kelchner 2000; Schlaepfer et al. 2008). Simple Indel Coding (SIC) was applied to gaps using the program FastGap 1.2 (Borchsenius 2009) and following the method of Simmons and Ochoterena (2000).

Haplotype networks:

Implementing three nucleotide substitution schemes in jModelTest 2.1.3 (Guidon & Gascuel 2003; Darriba et al. 2012), the best fitting model of nucleotide evolution was F81 for all regions based on the Bayesian Information Criterion (BIC). Thus, all chloroplast regions could be combined for further analyses. We used TCS 1.21 (Clement et al. 2000), a program using statistical parsimony to calculate a distance matrix for all pairwise comparisons of haplotypes, to estimate gene genealogies.

GenBank Sequences:

**trnV-atpE:** BankIt1918451, KX242743 to KX242966

**trnK-rps16:** BankIt1919233, KX242967 to KX243190

**rpl36-rps8:** BankIt1916902, KX227145 to KX227368

**trnH-psbA:** BankIt1916775, KX214780 to KX215003

**References:**

Borchsenius F. 2009.  FastGap 1.2. Department of Biosciences, Aarhus University, Denmark. Published online at <http://www.aubot.dk/FastGap_home.htm>

Clement M, Posada D and Crandall K. 2000. TCS: a computer program to estimate gene genealogies. Molecular Ecology 9(10): 1657-1660.

Darriba D, Taboada GL, Doallo R, Posada D. 2012. jModelTest 2: more models, new heuristics and parallel computing. [Nature Methods 9(8), 772.](http://www.nature.com/nmeth/journal/v9/n8/full/nmeth.2109.html)

Huelsenbeck, J. P. and F. Ronquist. 2001. MRBAYES: Bayesian inference of phylogeny. Bioinformatics 17:754-755.

Geneious version 5.6.6 created by Biomatters. Available from <http://www.geneious.com/>

Guindon S and Gascuel O. 2003. A simple, fast and accurate method to estimate large phylogenies by maximum-likelihood. Systematic Biology 52: 696-704.

Kelchner SA. 2000. The evolution of non-coding chloroplast DNA and its application in plant systematics. Annals of the Missouri Botanical Garden, 87, 482–498.

Kress WJ, Wurdack KJ, Zimmer EA, Weigt LA, Janzen DH. 2005. Use of DNA barcodes to identify flowering plants. *Proceedings of the National Academy of Sciences, USA*, 102, 8369–8374.

Sequencher® version 5.1 sequence analysis software, Gene Codes Corporation, Ann Arbor, MI USA. Available from [http://www.genecodes.com](http://www.genecodes.com/)

Simmons MP and Ochoterena H. 2000. Gaps as characters in sequence-based phylogenetic analyses. Syst Biol. 49 (2): 369-81.
