# Supplementary material for: Population genetics and adaptation to climate along elevation gradients in invasive Solidago canadensis
Source: PLoS One. 2017 Sep 28;12(9):e0185539. doi: 10.1371/journal.pone.0185539 (PMC5619793; doi:10.1371/journal.pone.0185539)
Supplement: S3 File — (DOCX) [file pone.0185539.s005.docx]

**S3 File: Bayesian Model Setup**

Environmental covariates:

Environmental covariates were calculated as described in the main text. In the following analyses, we divided degree-days by 100 and radiation by 1000 so that the parameters representing their effects would be more easily distinguishable from zero (eg. β = 1.5, rather than β = 0.015).

**Ranges for environmental distances (garden - home) were thus:**

**DD:** Degree-days/100: -14.52 to 1.986

**P:** Precipitation (cm/mo): -6.07 to 7.96

**JT:** Mean July temperature (°C): -8.03 to 0.67

**F:** Frost index: -22 to 18.42

**Yrad:** Mean global potential shortwave radiation, full year (MJ/m^2^/day): -2 to 8.17

**Mrad:** Mean global potential shortwave radiation, March (MJ/m^2^/day): -3 to 10.9

**PF:** Growing season precipitation days: -7 to 22

**S:** Slope (degrees inclination): -14.6 to 24.6

**A:** Aspect (compass degrees): -283 to 135.9

Survival model details

Survival (Surv) is coded as 0 (dead) or 1 (alive). It can thus be modeled as following a Bernoulli distribution with a probability connected via a logit link to covariates X:

$$Surv\sim Bern\left( \theta\right) logit\left( \theta\right)=X\rho+\varepsilon\varepsilon\sim N(0,\sigma^{2})$$

With only fixed effects (*β*):

$$P\left( \beta,\sigma^{2} | Surv \right)\propto P\left( Surv | \beta,\sigma^{2} \right)P\left( \beta\right)P\left( \sigma^{2} \right)=P\left( Surv | \theta\right)P\left( \beta\right)P\left( \sigma^{2} \right)$$

With fixed and random effects (*β & α*):

$$P\left( \beta,\sigma^{2},C,S^{2} | Surv \right)\propto P\left( Surv | \beta,\alpha,\sigma^{2} \right)P\left( \alpha| C,S^{2} \right)P\left( \beta\right)P\left( \sigma^{2} \right)P\left( C \right)P\left( S^{2} \right)=\left( Surv | \theta\right)P\left( \alpha| C,S^{2} \right)P\left( \beta\right)P\left( \sigma^{2} \right)P\left( C \right)P\left( S^{2} \right)$$

HGR model details:

HGR is a continuous measure and can be modeled using a linear model with Normal error:

$HGR\sim N\left( \mu,\sigma^{2} \right) \mu=X\rho$

With only fixed effects (*β*):

$$P\left( \beta,\sigma^{2} | HGR \right)\propto P\left( HGR | \beta,\sigma^{2} \right)P\left( \beta\right)P\left( \sigma^{2} \right)$$

With fixed and random effects (*β & α*):

$$P\left( \beta,\sigma^{2},C,S^{2} | HGR \right)\propto P\left( HGR | \beta,\alpha,\sigma^{2} \right)P\left( \alpha| C,S^{2} \right)P\left( \beta\right)P\left( \sigma^{2} \right)P\left( C \right)P\left( S^{2} \right)$$

Flower model details:

Flower number is best modeled as following a Poisson distribution with the Poisson mean/variance parameter connected via a log link to covariates X:

$$Flwr\sim Poisson\left( \lambda\right) \ln\left( \lambda\right)=X\rho+\varepsilon\varepsilon\sim N(0,\sigma^{2})$$

With only fixed effects (*β*):

$$P\left( \beta,\sigma^{2} | Flwr \right)\propto P\left( Flwr | \beta,\sigma^{2} \right)P\left( \beta\right)P\left( \sigma^{2} \right)=P\left( Flwr | \lambda\right)P\left( \beta\right)P\left( \sigma^{2} \right)$$

With fixed and random effects (*β & α*):

$$P\left( \beta,\sigma^{2},C,S^{2} | Flwr \right)\propto P\left( Flwr | \beta,\alpha,\sigma^{2} \right)P\left( \alpha| C,S^{2} \right)P\left( \beta\right)P\left( \sigma^{2} \right)P\left( C \right)P\left( S^{2} \right)=\left( Flwr | \lambda\right)P\left( \alpha| C,S^{2} \right)P\left( \beta\right)P\left( \sigma^{2} \right)P\left( C \right)P\left( S^{2} \right)$$

Conjugate priors:

For all of these models we can take advantage of the properties of certain probability distributions when choosing priors. The likelihood often takes the form of a Normal distribution – this is most easily seen for the likelihood in the HGR model. Most of the fixed and random effect parameters could take either positive or negative values, and thus it makes sense to give them Normal priors. The product of two Normal distributions is another Normal distribution:

$$N\left( y|\mu,\sigma^{2} \right)N\left( \mu|\theta_{0},\tau_{0}^{2} \right)=N\left( \mu| \theta_{1},\tau_{1}^{2} \right)$$

$$\theta_{1}=\tau_{1}^{2}\left( \frac{\theta_{0}}{\tau_{0}^{2}}+\frac{n\bar{y}}{\sigma^{2}} \right) \tau_{1}^{2}=\left( \frac{1}{\tau_{0}^{2}}+\frac{n}{\sigma^{2}} \right)^{-1}$$

Variance parameters must be greater than zero. In this case, it makes sense to use an inverse gamma distribution, since the product of a Normal distribution and an inverse gamma distribution is an inverse gamma distribution:

$$N\left( y|\mu,\sigma^{2} \right)IG\left( \sigma^{2}|\alpha,\beta\right)=IG\left( \sigma^{2}|S_{1},S_{2} \right)$$

$$S_{1}=\frac{n}{2}+\alpha+1 S_{2}=\beta+\frac{1}{2}\sum_{i=1}^{n} {(y_{i}-\mu)}^{2}$$

Thus, when these relationships apply, new values can be drawn directly from the posterior at each step of the Gibbs sampler.

Choosing priors:

In all models, “*β_1_*” is the intercept of the linear equation, representing essentially a mean response without the modifying effects of site, clone, etc. For HGR, *β_1_*= 1 would represent an average growth rate of 1 cm/day. This is a reasonable expected mean for *Solidago*, which often reaches 1-1.5 m over 3-4 months. Plants do not lose height in the initial period of growth unless damaged (rare), and rarely grow more than 3 cm/day. Therefore in the HGR model, the prior for *β_1_* was a normal distribution with mean= 1 and standard deviation = 1 (hereafter N(1,1)). In the survival model, logit^-1^(*β_1_*) represents mean survival probability. Most individuals survived, so the prior for *β_1_*  was N(2,1). This yields a mean survival of 0.881, with 95% of values between 0.5 and 0.982. In the flowering model, e*^β1^* represents mean expected flowers. The average number of total flower-buds or mature flowers was lower in 2013 and higher in 2014. For total number of flower buds, we assigned *β_1_* ~ N(0.5,1) for 2013 (mean 1.65, 95% range 0.22 – 12.18), and N(1.5,1) for 2014 (mean 4.48, 95% range 0.61 – 33.11). For total mature flowers, we assigned *β_1_* ~ N(-1.5,1.2) for 2013 (mean 0.22, 95% range 0.02 – 2.46), and N(-1,1.2) for 2014 (mean 0.37, 95% range 0.03 – 4.06).

For the variance parameter (σ^2^) of the linear equation, we assigned an inverse gamma (IG) prior, which has 2 parameters and is always >0. In the HGR model, we assigned IG(2.5,1.215), which has a mean of 0.81 and a variance of 1.31. In the survival model, we assigned IG(2.6,0.4), which has a mean of 0.25 and a variance of 0.104. In the flowering model, we assigned we assigned IG(2.15,0.184), which has a mean of 0.16 and a variance of 0.1707.

We assigned broad priors for effect of site to reflecting our expectation that higher sites would have lower growth and flowering, but might not have lower survival. For the effects of low, medium, and high sites on HGR, we assigned priors N(0.2,0.7), N(0,0.7), and N(-0.2,0.7). For the effects of low, medium, and high sites on flowering, we assigned priors N(0.3,1), N(0,1), and N(-1.5,1) for total flowers in 2013; N(0.4,1), N(0,1), and N(-0.5,1) for total flowers in 2014; N(1.5,1), N(0,1), and N(-1,1) for mature flowers in 2013; N(2.5,1), N(1,1), and N(-1.5,1) for mature flowers in 2014. For the effects of low, medium, and high sites on survival, we assigned priors N(0,0.8).

For climate variables, population ID, and initial size, it was unclear whether they would affect performance positively or negatively. Therefore, we assigned Normal priors centered around zero for all these variables, usually with a standard deviation = 0.8. For clone effects, we expect that individual clones might have higher or lower performance than the average, and we assume that clone effects are drawn from a normal distribution with mean C and variance S. For models including clone effects, we needed to assign priors for C and S. C is likely to be near 0. For all analyses, we used priors: C~N(0, 0.7), and S ~IG(2.1,0.22).

Gibbs Sampler setup:

Starting values for parameters were drawn from uniform distributions reflecting a wide range of plausible values. For instance, in HGR analyses we usually used: intercept 0.2 to 2, site effects -0.3 to 0.3, climate and size effects -0.5 to 0.5, sigma-squared 0.05 to 0.3, clone effects -0.4 to 0.4, C -0.2 to 0.2, and S 0.05 to 0.4. The spread of the uniform distributions were occasionally reduced in the more complex models where randomly choosing high values or low values for all parameters could produce divide-by-zero errors or other computational problems. As the Gibbs sampler runs, the estimated parameter values quickly diverge from the initial values, trending toward the true value.

In tests, we found that the Gibbs Sampler tended to converge quickly, so for all models not including clone we used a series of 10,000 Gibbs steps, with a 2,000 step burn-in. For the more complex models including clone effects we used a series of 30,000 Gibbs steps, with a 3,000 step burn-in. The value of each parameter at each Gibbs step is saved in an output matrix.

For the HGR model without random effects, *β* and *σ^2^* could be updated simply and sequentially using the conjugate likelihood-prior pairs. For each Gibbs step *t*:

$$\beta_{(t)}\sim f\left( \beta| RGR,\sigma_{(t-1)}^{2} \right)=N\left( RGR | {X\beta}_{(t-1)},\sigma_{(t-1)}^{2} \right)N\left( \beta|b_{m},b_{v} \right)$$

$$\sigma_{(t)}^{2}\sim f\left( \sigma^{2} | RGR,\beta_{(t)} \right)=N\left( RGR | {X\beta}_{(t)},\sigma_{(t-1)}^{2} \right)IG\left( \sigma^{2}|s_{1},s_{2} \right)$$

where b_m_ and b_v_ are the prior means and variances for the betas, and s_1_,s_2_ are the prior inverse-gamma parameters for σ^2^.

For the model with random effects, there are two more steps to the update:

$${(\beta}_{\left( t \right)},\alpha_{(t)})\sim f\left( \beta,\alpha| RGR,\sigma_{(t-1)}^{2},C_{(t-1)},S_{(t-1)}^{2} \right)=N\left( RGR | X[\beta_{(t-1)},\alpha_{(t-1)}],\sigma^{2} \right)N\left( \alpha_{(t)} | C_{(t-1)},S_{(t-1)}^{2} \right)N\left( \beta|b_{m},b_{v} \right)$$

$$C_{(t)}\sim f\left( C | \alpha_{(t)},\sigma_{(t-1)}^{2},S_{(t-1)}^{2} \right)=N\left( \alpha_{(t)} | C_{(t-1)},S_{(t-1)}^{2} \right)N\left( C|c_{m},c_{v} \right)$$

$$S_{(t)}^{2}\sim f\left( S^{2} | \alpha_{(t)},\sigma_{(t-1)}^{2},C_{(t)} \right)=N\left( \alpha_{(t)} | C_{(t)},S_{(t-1)}^{2} \right)IG\left( S^{2}|s_{3},s_{4} \right)$$

$$\sigma_{(t)}^{2}\sim f\left( \sigma^{2} | RGR,{\alpha_{(t)}\beta}_{\left( t \right)},C_{(t)},S_{(t)}^{2} \right)=N\left( RGR | {X[\beta}_{\left( t \right)},\alpha_{(t)}],\sigma_{(t-1)}^{2} \right)IG\left( \sigma^{2}|s_{1},s_{2} \right)$$

where c_m_ and c_v_ are the prior mean and variance for C, and s_3_,s_4_ are the prior inverse-gamma parameters for S^2^.

For the survival model or flowering model, where there is a logit or log link between the linear equation and the response variable, this linkage cannot be captured just by conjugate priors and likelihoods. In the following equations, *γ* =logit(*θ*) or log(λ), and Y = survival or # of flowers, respectively. First, we propose a vector of *γ’s* based on the current values of *α*, *β*, and *σ^2^*:

$$\gamma_{(t-1)}\sim N(X\left[ \beta_{\left( t-1 \right)},\alpha_{\left( t-1 \right)} \right],S_{(t-1)}^{2})$$

Without random effects, we first update *β* and *σ^2^*:

$$\beta_{(t)}\sim f\left( \beta| \gamma_{(t-1)},\sigma_{(t-1)}^{2} \right)=N\left( \gamma_{(t-1)} | {X\beta}_{(t-1)},\sigma_{(t-1)}^{2} \right)N\left( \beta|b_{m},b_{v} \right)$$

$$\sigma_{(t)}^{2}\sim f\left( \sigma^{2} | \gamma_{(t-1)},\beta_{(t)} \right)=N\left( \gamma_{(t-1)} | {X\beta}_{(t)},\sigma_{(t-1)}^{2} \right)IG\left( \sigma^{2}|s_{1},s_{2} \right)$$

Then we draw a new vector of proposed *γ* values such that:

$$\gamma^{*}\sim N(X\left[ \beta_{\left( t \right)},\alpha_{\left( t \right)} \right],\sigma_{(t)}^{2})$$

For each individual *i,* if the log-likelihood of the data given *γ_i_^*^* is greater than the log-likelihood of the data given *γ_(i,t-1)_* , then the new value is accepted. If the log-likelihood with *γ_i_^*^* is less than the log-likelihood *γ_(i,t-1)_* then the probability of acceptance is equal to ln(p(*Y_i_*| *γ_i_^*^*))/ln(p(*Y_i_*| *γ_(i,t-1)_*)).

For the survival or flowering models with random effects,

$${(\beta}_{\left( t \right)},\alpha_{(t)})\sim f\left( \beta,\alpha| \gamma_{(t-1)},\sigma_{(t-1)}^{2},C_{(t-1)},S_{(t-1)}^{2} \right)=N\left( \gamma_{(t-1)} | X[\beta_{(t-1)},\alpha_{(t-1)}],\sigma^{2} \right)N\left( \alpha_{(t)} | C_{(t-1)},S_{(t-1)}^{2} \right)N\left( \beta|b_{m},b_{v} \right)$$

$$C_{(t)}\sim f\left( C | \gamma_{(t-1)},S_{(t-1)}^{2} \right)=N\left( \alpha_{(t)} | C_{(t-1)},S_{(t-1)}^{2} \right)N\left( C|c_{m},c_{v} \right)$$

$$S_{(t)}^{2}\sim f\left( S^{2} | \gamma_{(t-1)},\alpha_{(t)},C_{(t)} \right)=N\left( \alpha_{(t)} | C_{(t)},S_{(t-1)}^{2} \right)IG\left( S^{2}|s_{3},s_{4} \right)$$

$$\sigma_{(t)}^{2}\sim f\left( \sigma^{2} | \gamma_{(t-1)},\beta_{\left( t \right)},\alpha_{(t)} \right)=N\left( \gamma_{(t-1)} | {X[\beta}_{\left( t \right)},\alpha_{(t)}],\sigma_{(t-1)}^{2} \right)IG\left( \sigma^{2}|s_{1},s_{2} \right)$$

The *γ* values are updated as described above.

Calculating mean and spread of posterior for each parameter:

The output of the Gibbs sampler after the burn-in period is thinned to remove autocorrelation. We thinned to every 20^th^ step. From this reduced sequence of values, we calculated the mean (best estimate), standard deviation, and 95% credible interval (quantiles 0.025-0.975). In test runs, if the true parameter value (from which we simulated the test data) fell within the 95% credible interval, we considered that the model estimated that value appropriately.

Calculating predictive loss:

As mentioned in the main text, where *m* is the model variant in question:

$$D_{m}=\sum_{i=1}^{n} \left( E\left[ y_{i} | \vec{y} \right]-y_{i} \right)^{2} +\sum_{i=1}^{n} var\left[ y_{i} | \vec{y} \right]$$

This can be calculated based on the output of the Gibbs sampler. For each of the thinned post-burn-in Gibbs steps, we used the values of the parameters to generate a predicted y. For instance:

$${RGR}_{pred 2020}\sim N(X\beta_{2020},\sigma_{2020}^{2})$$

Then, we calculated *D_m_* using:

$$E\left[ y_{i} | \vec{y} \right]=Mean\left( {RGR}_{pred} \right) var\left[ y_{i} | \vec{y} \right]=Var({RGR}_{pred})$$

Tests:

We tested all models with simulated data to make sure that parameters could be recovered and the predictive loss preferred the correct model. For instance, for the HGR model, we generated data using an intercept (*β_1_*) of 1, Chlara effect (*β_2_*) of 0.2, Jeli effect (*β_3_*) of 0, Zweierspitz effect (*β_4_*) of -0.2, and *σ^2^* of 0.2. The output of the Gibbs sampler for one run is shown in Figure 1, while Table 1 shows posteriors compared to the true parameter values. You can see that the true values fall within the 95% credible intervals.


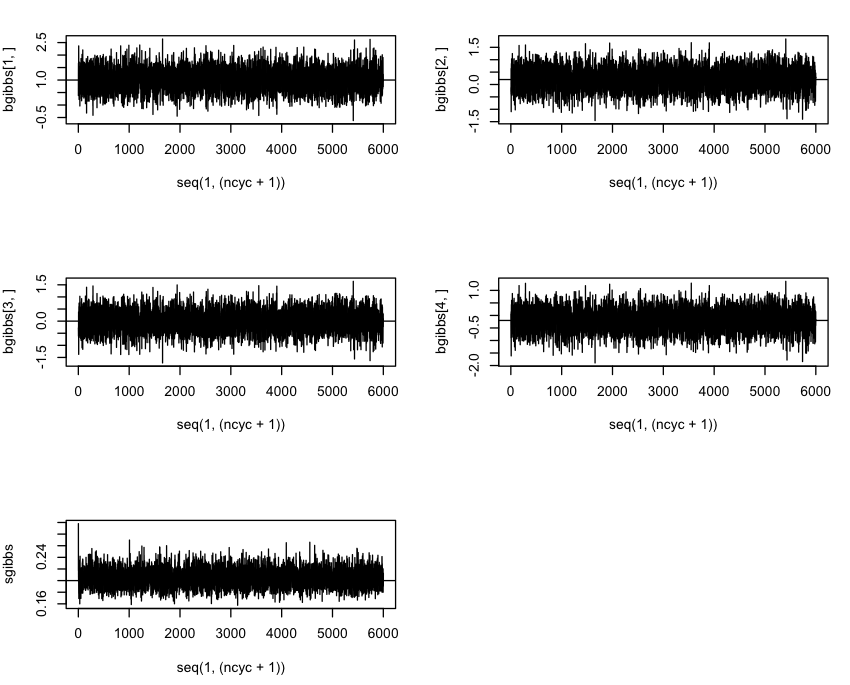


Figure A: Gibbs sampler output for betas and sigma-squared. Line indicates true parameter

value.

Table A: Estimate and credible interval for simulated data with true model

|  | **Estimate** | **True** | **Quantile 0.025** | **Quantile 0.975** |
| --- | --- | --- | --- | --- |
| ***β_1_*** | 0.99646 | 1 | 0.14645 | 1.87711 |
| ***β_2_*** | 0.23351 | 0.2 | -0.65101 | 1.12409 |
| ***β_3_*** | 0.02417 | 0 | -0.84480 | 0.86092 |
| ***β_4_*** | -0.17681 | -0.2 | -1.01465 | 0.66842 |
| ***σ^2^*** | 0.20493 | 0.2 | 0.17714 | 0.23534 |

Notice that the 95% CI’s for the site effect parameters overlap zero. So this is not enough to determine whether a parameter contributes importantly to model fit. We tested a version of the model in which the data generated as above was fit with a model that included not only site effects, but also effects of overall climate distance, degree-day distance, and precipitation distance (*β_5_*- *β_7_*). For instance, for 3 different sets of simulated data runs, the Predictive Loss for the correct simpler model was 138.8332, 132.8469, and 120.6224, while for the more complex model it was 139.3364, 134.0372, and 121.5985. Predictive loss is almost always lower for the (correct) simpler model. In addition, the estimates of the non-existent effects are usually much closer to zero than are the important effects.

Table B: Model test results

|  | **Estimate** | **True** | **Quartile 0.025** | **Quartile 0.975** |
| --- | --- | --- | --- | --- |
| ***β_1_*** | 1.06599 | 1 | 0.25365 | 1.97662 |
| ***β_2_*** | 0.19867 | 0.2 | -0.68782 | 0.98065 |
| ***β_3_*** | -0.00978 | 0 | -0.85762 | 0.79803 |
| ***β_4_*** | -0.29365 | -0.2 | -1.19028 | 0.54524 |
| ***β_5_*** | -0.00395 | - | -0.06780 | 0.05968 |
| ***β_6_*** | -0.00041 | - | -0.00321 | 0.00247 |
| ***β_7_*** | -0.00001 | - | -0.00229 | 0.00214 |
| ***σ^2^*** | 0.19527 | 0.2 | 0.16844 | 0.22338 |
