# Supplementary material for: Population genetics and adaptation to climate along elevation gradients in invasive Solidago canadensis
Source: PLoS One. 2017 Sep 28;12(9):e0185539. doi: 10.1371/journal.pone.0185539 (PMC5619793; doi:10.1371/journal.pone.0185539)
Supplement: S4 File — (DOCX) [file pone.0185539.s006.docx]

**S4 File: Additional population genetic structure results**

**
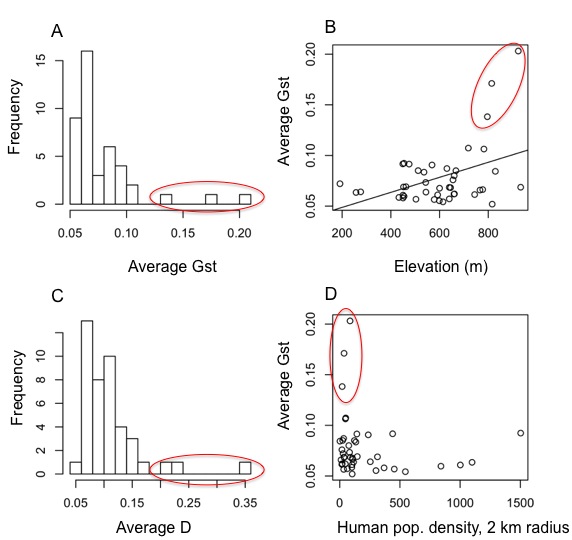
**

**Figure A:** (A & C) Histograms of average Gst and D based on microsatellite loci for each population. Notice that three populations (HH1, HL2, & MM2) are more highly differentiated that the rest. These populations are from high elevation areas (B), with low human population density (D).

**
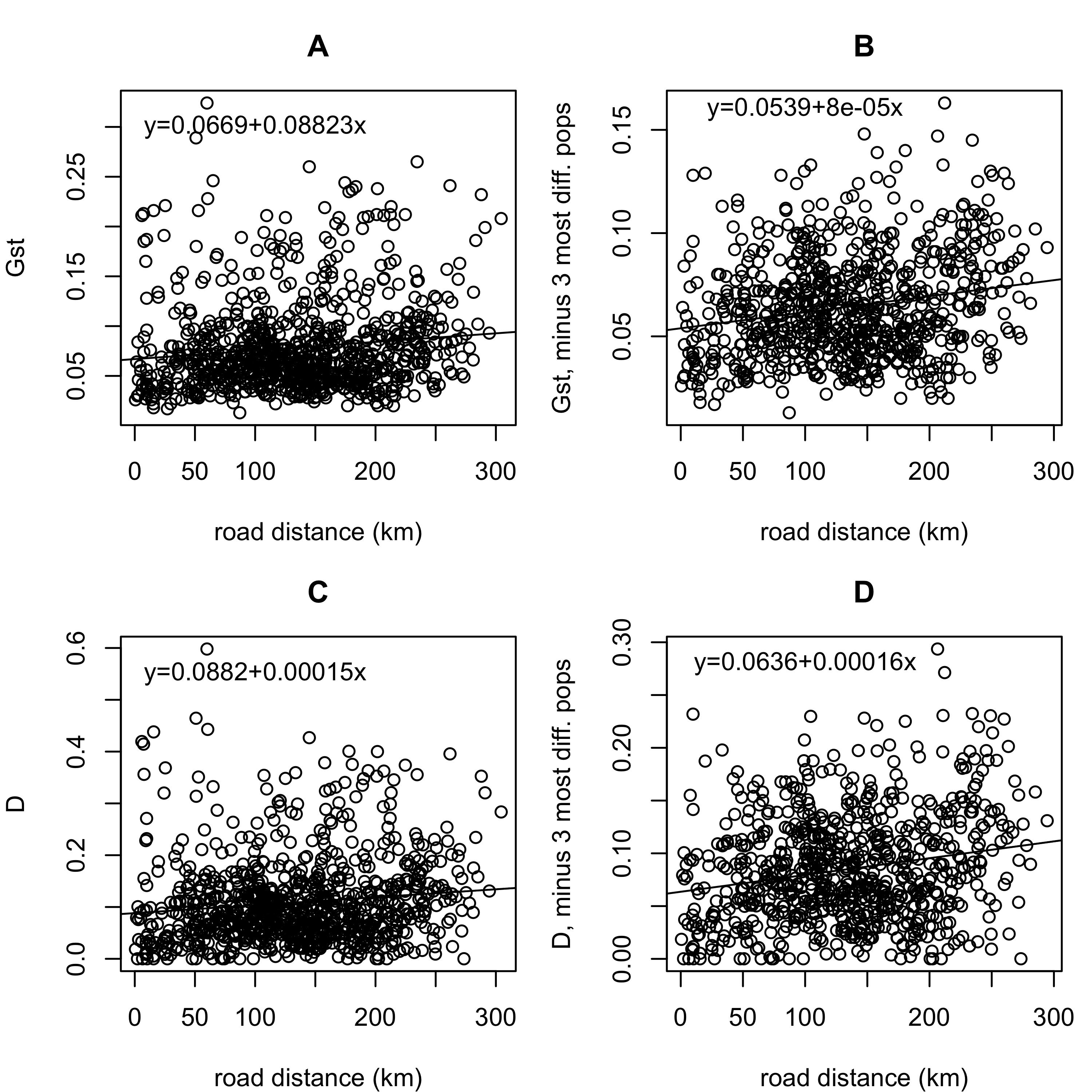
**

**Figure B:** (A & B) Pairwise nuclear G_ST_ versus road distance either with (A) or without (B) the three most differentiated populations. (C & D) Pairwise nuclear D versus road distance either with (C) or without (D) the three most differentiated populations.


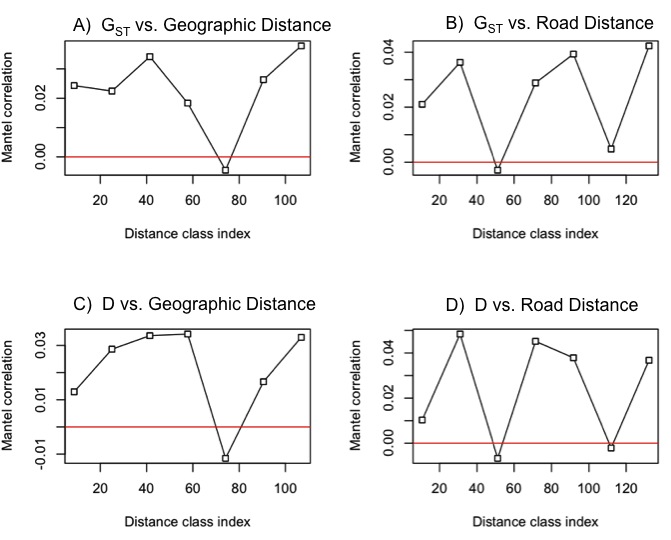


**Figure C:**  Mantel correlograms for all populations. Distance class in meters for geographic (straight line) distance or distance by road.


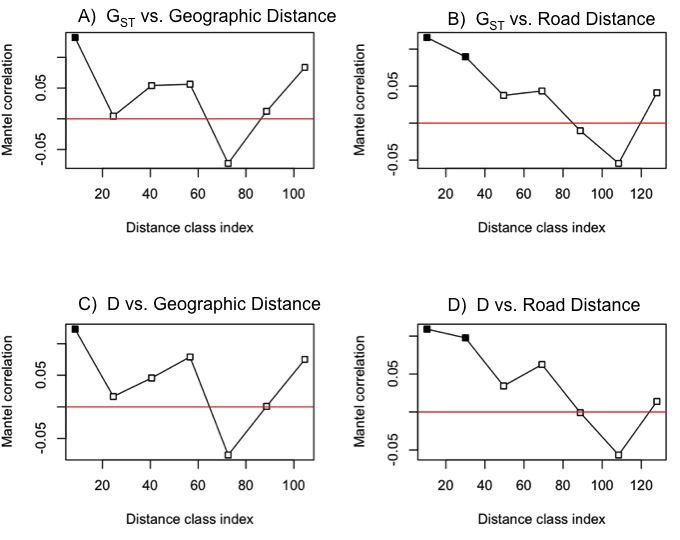


**Figure D:** Mantel correlograms for all but the three most differentiated populations. Distance class in meters for geographic (straight line) distance or distance by road.


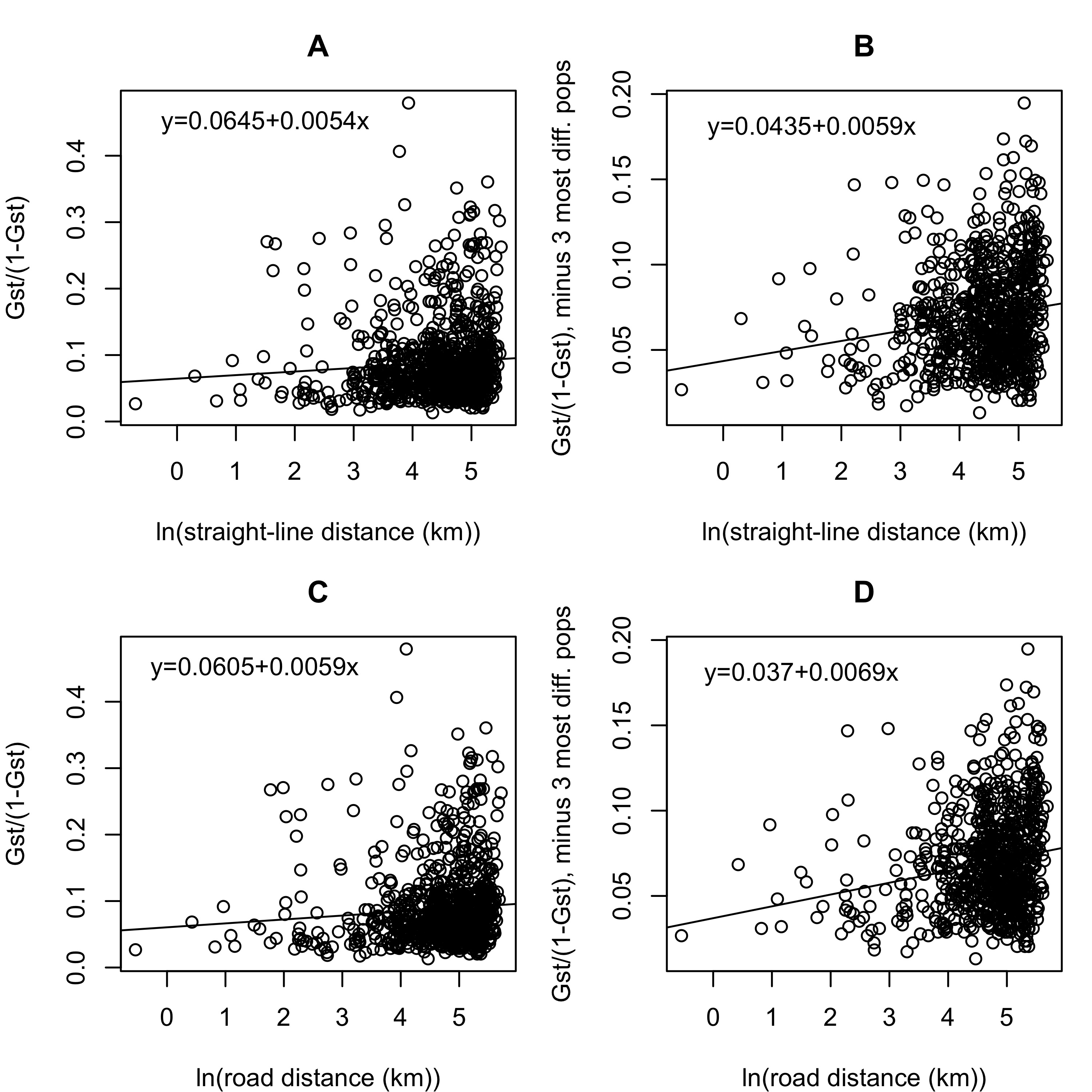


**Figure E:** Rousset test for isolation by distance. If populations form a 2-D lattice of "stepping stones", we expect the relationship between G_ST_/(1- G_ST_) and ln(distance) to be linear. (A & B) G_ST_/(1- G_ST_) versus ln(straight-line distance), either with (A; R^2^ =0.005) or without (B; R^2^=0.026) the three most differentiated populations. (C & D ) G_ST_/(1- G_ST_) versus ln(distance-by-road) either with (C; R^2^=0.006) or without (D; R^2^ = 0.034) the three most differentiated populations.

**Table A:** Linear regression of various differentiation measures against distance class, as shown in panels C & D of Figure 1 in text.

|  | Intercept | Distance Class parameter | P-value (Distance Class effect) | Adjusted R-squared |
| --- | --- | --- | --- | --- |
| Chloroplast G_ST_ | 0.399 | 0.0006626 | 0.000419 | 0.3994 |
| Chloroplast N_ST_ | 0.449 | 0.000560 | 0.0357 | 0.1422 |
| Microsatellite G_ST_ | 3.761e-02 | 1.418e-04 | 0.0025 | 0.3047 |
| Pollen:Seed | 1.3736 | 0.0006295 | 0.000875 | 0.3618 |

**Table B:**  MMRR analysis of IBE vs. IBD, all populations. * - significant at p=0.05; ^ - significant at p=0.1.

| Genetic | Environ | Geog | model r-sq | Env coeff (p) | Geo coeff (p) |
| --- | --- | --- | --- | --- | --- |
| G_ST_ | Climate PCA | Road | 0.031 | -4.736e-03  (0.257) | 9.430e-08  (0.034*) |
| G_ST_ | Degree-day | Road | 0.016 | -1.902e-07  (0.927) | 8.826e-08  (0.044*) |
| G_ST_ | Precip | Road | 0.02 | -1.097e-06  (0.542) | 9.184e-08  (0.049*) |
| G_ST_ | Jul Temp | Road | 0.016 | -1.570e-06  (0.966) | 8.759e-08  (0.032*) |
| G_ST_ | GS Frost | Road | 0.025 | 7.125e-04  (0.363) | 8.005e-08  (0.065^) |
| G_ST_ | Annual Radiation | Road | 0.016 | -4.924e-07  (0.862) | 8.698e-08  (0.048*) |
| G_ST_ | Mar Radiation | Road | 0.016 | -3.368e-07  (0.880) | 8.667e-08  (0.054^) |
| G_ST_ | Precip Fre. | Road | 0.016 | -4.506e-06  (0.993) | 8.705e-08  (0.112) |
| G_ST_ | Climate PCA | Geo | 0.029 | -4.722e-03  (0.279) | 1.057e-07  (0.046*) |
| D | Climate PCA | Road | 0.022 | -5.702e-03  (0.462) | 1.638e-07  (0.036*) |
| G_ST_ | GS Frost | Geo | 0.023 | 7.132e-04  (0.393) | 8.782e-08  (0.097^) |
| D | GS Frost | Road | 0.026 | 1.410e-03  (0.306) | 1.412e-07  (0.075^) |

**Table C:** MMRR analysis of IBE vs. IBD, minus 3 most differentiated populations. * - significant at p=0.05; ^ - significant at p=0.1

| Genetic | Environ | Geog | model r-sq | Env coeff (p) | Geo coeff (p) |
| --- | --- | --- | --- | --- | --- |
| G_ST_ | Climate PCA | Road | 0.049 | -1.797e-03 (0.432) | 8.164e-08 (0.002*) |
| G_ST_ | Degree-day | Road | 0.042 | -2.297e-07  (0.857) | 7.987e-08  (0.001*) |
| G_ST_ | Precip | Road | 0.042 | -2.817e-07  (0.736) | 7.976e-08  (0.003*) |
| G_ST_ | Jul Temp | Road | 0.042 | -3.569e-06  (0.905) | 7.969e-08  (0.001*) |
| G_ST_ | GS Frost | Road | 0.044 | 2.151e-04 (0.683) | 7.498e-08 (0.001*) |
| G_ST_ | Annual Radiation | Road | 0.044 | -6.098e-07  (0.680) | 7.858e-08  (0.001*) |
| G_ST_ | Mar Radiation | Road | 0.044 | -4.782e-07  (0.665) | 7.802e-08  (0.001*) |
| G_ST_ | Precip Fre. | Road | 0.042 | -5.529e-05  (0.871) | 7.9345e-08  (0.004*) |
| G_ST_ | Climate PCA | Geo | 0.036 | -1.722e-03  (0.471) | 8.225e-08  (0.004*) |
| D | Climate PCA | Road | 0.043 | -8.864e-04 (0.868) | 1.620e-07 (0.002*) |
| G_ST_ | GS Frost | Geo | 0.032 | 2.384e-04  (0.627) | 7.404e-08  (0.003*) |
| D | GS Frost | Road | 0.048 | 5.92e-04 (0.541) | 1.514e-07 (0.002*) |
