# Supplementary material for: Population genetics and adaptation to climate along elevation gradients in invasive Solidago canadensis
Source: PLoS One. 2017 Sep 28;12(9):e0185539. doi: 10.1371/journal.pone.0185539 (PMC5619793; doi:10.1371/journal.pone.0185539)
Supplement: S5 File — (DOCX) [file pone.0185539.s007.docx]

**S5 File: Performance across sites**

Spring in 2013 had a relatively cool and wet spring, with planting only being possible at the end of May due to snow at higher elevations, followed by a relatively hot, dry summer. A heavy snowfall broke many stems and ended the growing season for the upper two sites in early October, but inflorescences continued to mature at 659 m until mid-November, when plants were damaged by a windstorm. Spring in 2014 was early and warm, with plants beginning to bolt by March 28 at 659 m, and snow disappearing at the upper sites by early April, but the summer was relatively cool and damp. Plants at all three sites were harvested at the end of September 2014.

Table A: Performance at each site in each year

|  | **2013** | | | **2014** | | |
| --- | --- | --- | --- | --- | --- | --- |
|  | **659 m** | **1253 m** | **1680 m** | **659 m** | **1253 m** | **1680 m** |
| **Survival** | 0.799 | 0.847 | 0.951 | 0.792 | 0.847 | 0.951 |
| **Mean HGR (cm/day)** | 1.27 | 1.12 | 0.77 | 1.58 | 1.04 | 0.46 |
| **Max HGR**  **(cm/day)** | 2.11 | 1.85 | 1.23 | 1.98 | 1.36 | 0.64 |
| **Mean total flowers** | 1.68 | 1.11 | 0.05 | 8.61 | 8.06 | 7.23 |
| **Max total flowers** | 5 | 6 | 2 | 22 | 22 | 26 |
| **Mean mature flowers** | 1.3 | 0 | 0 | 8.44 | 1.07 | 0 |
| **Max mature flowers** | 5 | 0 | 0 | 22 | 12 | 0 |


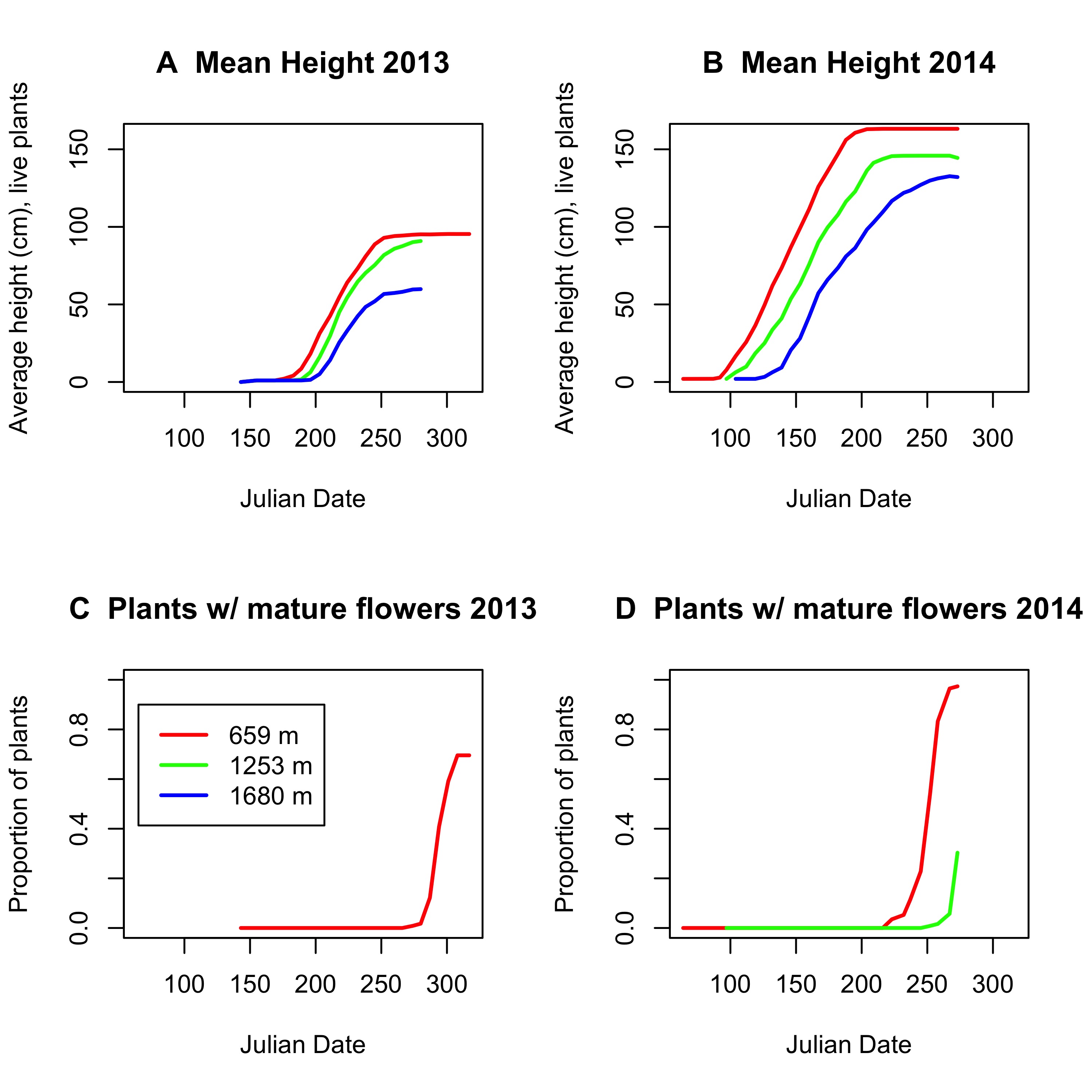


**Figure A:** (A) Average height of plants over time in the three common gardens in 2013. (B) Average height of plants over time in 2014. (C) Proportion of plants with mature inflorescences in 2013 - only those in lowest garden matured. (D) Proportion of plants with mature inflorescences in 2014 - only those in 2 lowest gardens matured.


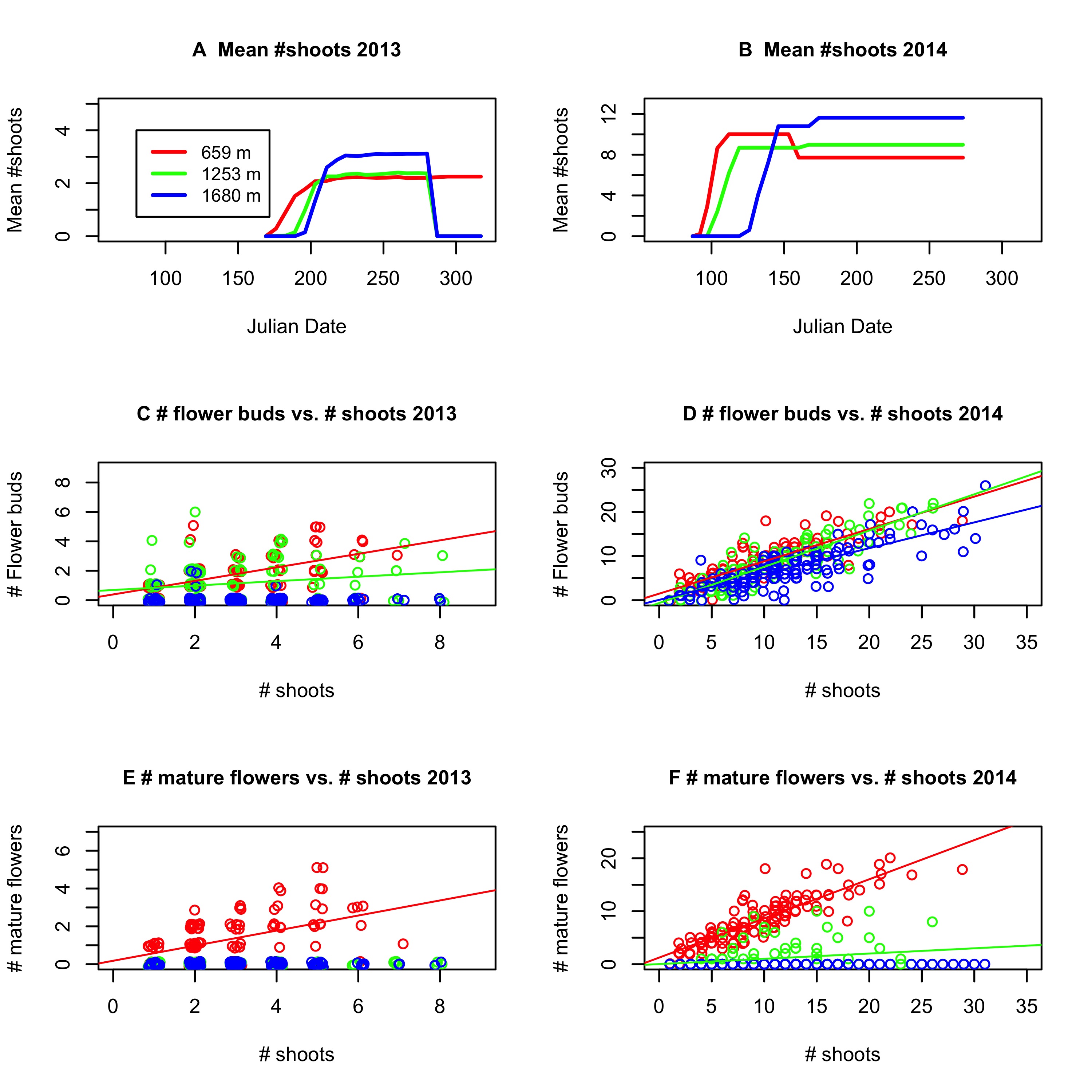


**Figure B:** (A) Average number of shoots over time in the three common gardens in 2014. (B) Average number of shoots over time in 2014. (C) Number of flower (inflorescence) buds vs. number of shoots in the three common gardens in 2013. (D) Number of flower (inflorescence) buds vs. number of shoots in 2014. (E) Number of mature flowers (inflorescences) vs. number of shoots in 2013. (F) Number of mature flowers (inflorescences) vs. number of shoots in 2014.

**
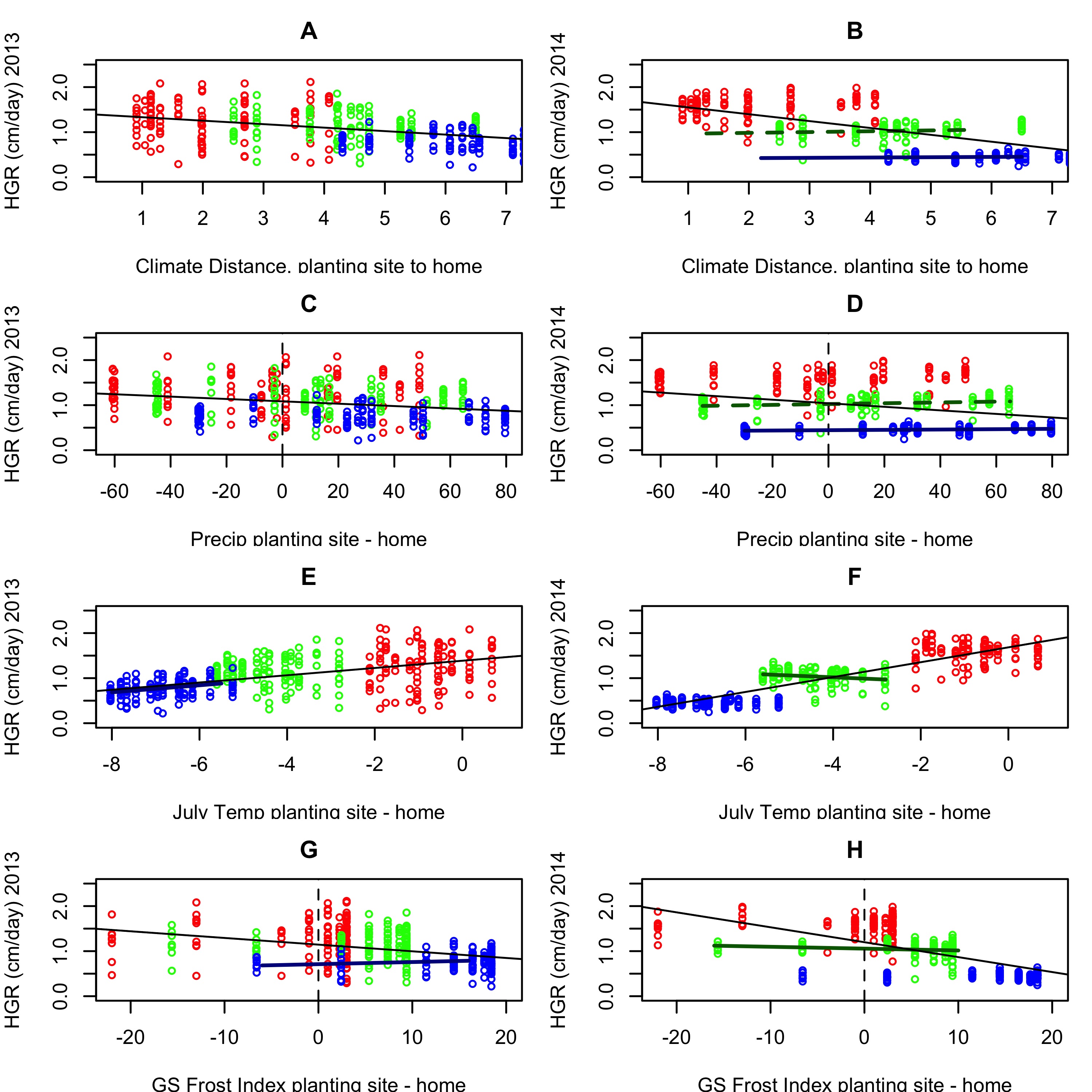
**

**Figure C:** Height growth rate (HGR) vs. various measures of climatic transfer distance in 2013 (left) and 2014 (right). (A & B) Overall climate distance based on PCA. (C & D) Precipitation distance, 2013. (E & F) July temperature distance. (G & H) Growing season frost index distance. Red dots - low elevation site. Green - mid elevation site. Blue - high elevation site. Thick colored lines indicate within-site relationships. Thin black lines indicate across-site relationships. Solid lines indicate statistically significant relationships. Dashed lines indicate marginally significant relationship (p<0.1).


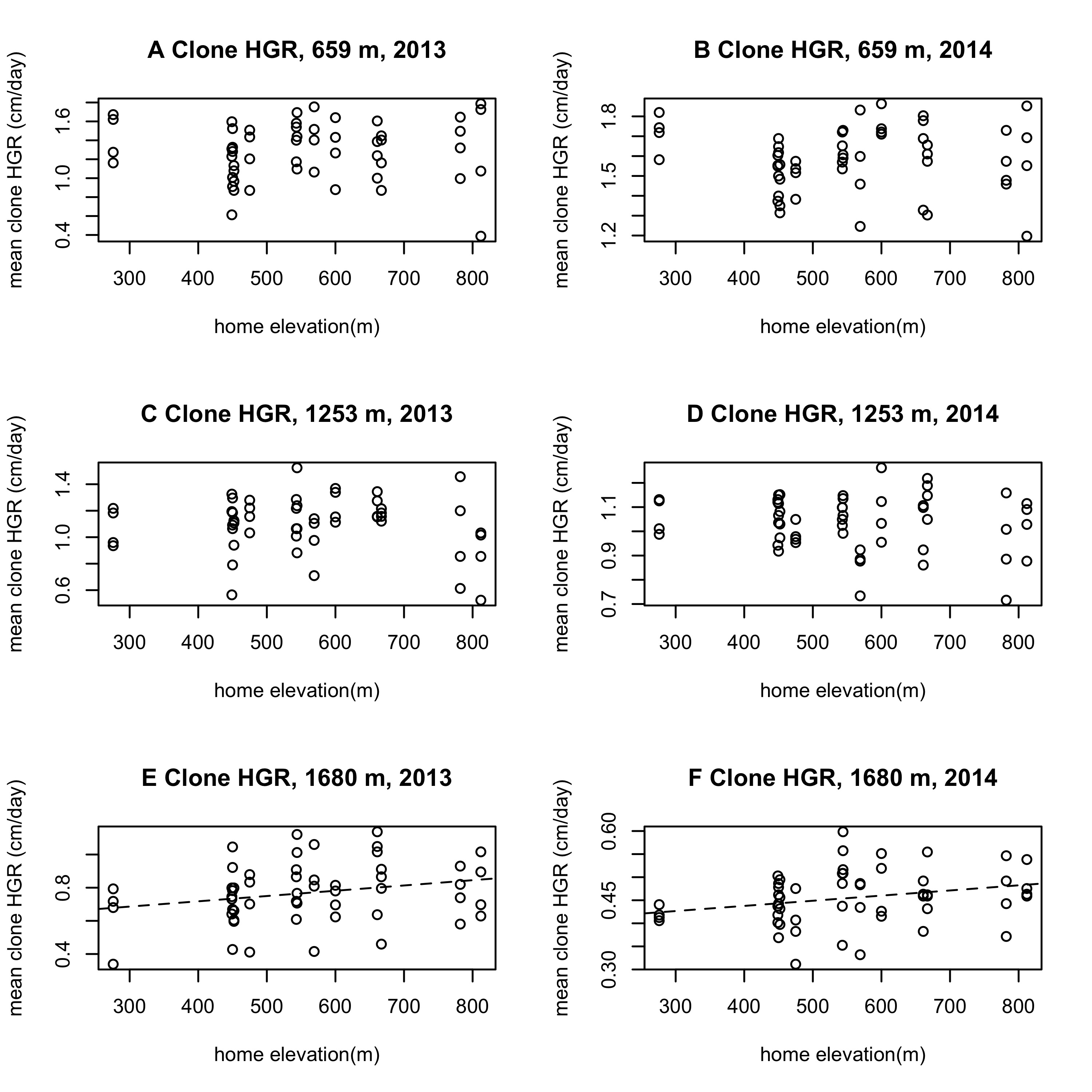


**Figure D:** Average clone growth rate at each site in each year. HGR is marginally positively correlated with home elevation at the highest site.


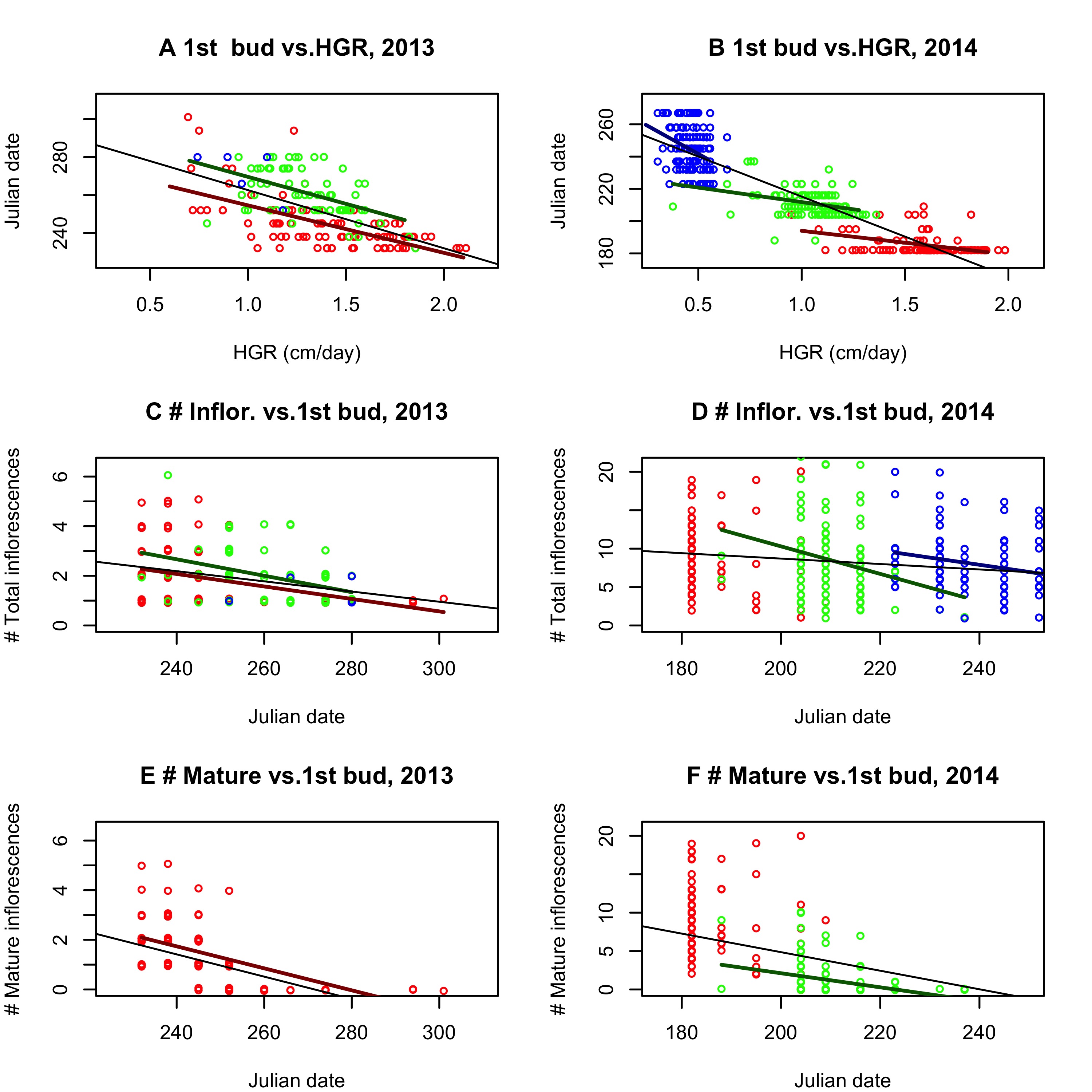


**Figure E:** Left panels depict 2013, right panels 2014. (A & B) Date first flower bud recorded vs. HGR. (C&D) Total number of flowers vs. date first flower bud formed. (E& F): Number of mature flowers vs. date first flower bud formed. Thick colored lines indicate within-site relationships. Thin black lines indicate across-site relationships. Solid lines indicate statistically significant relationships. Dashed lines indicate marginally significant relationship (p<0.1).


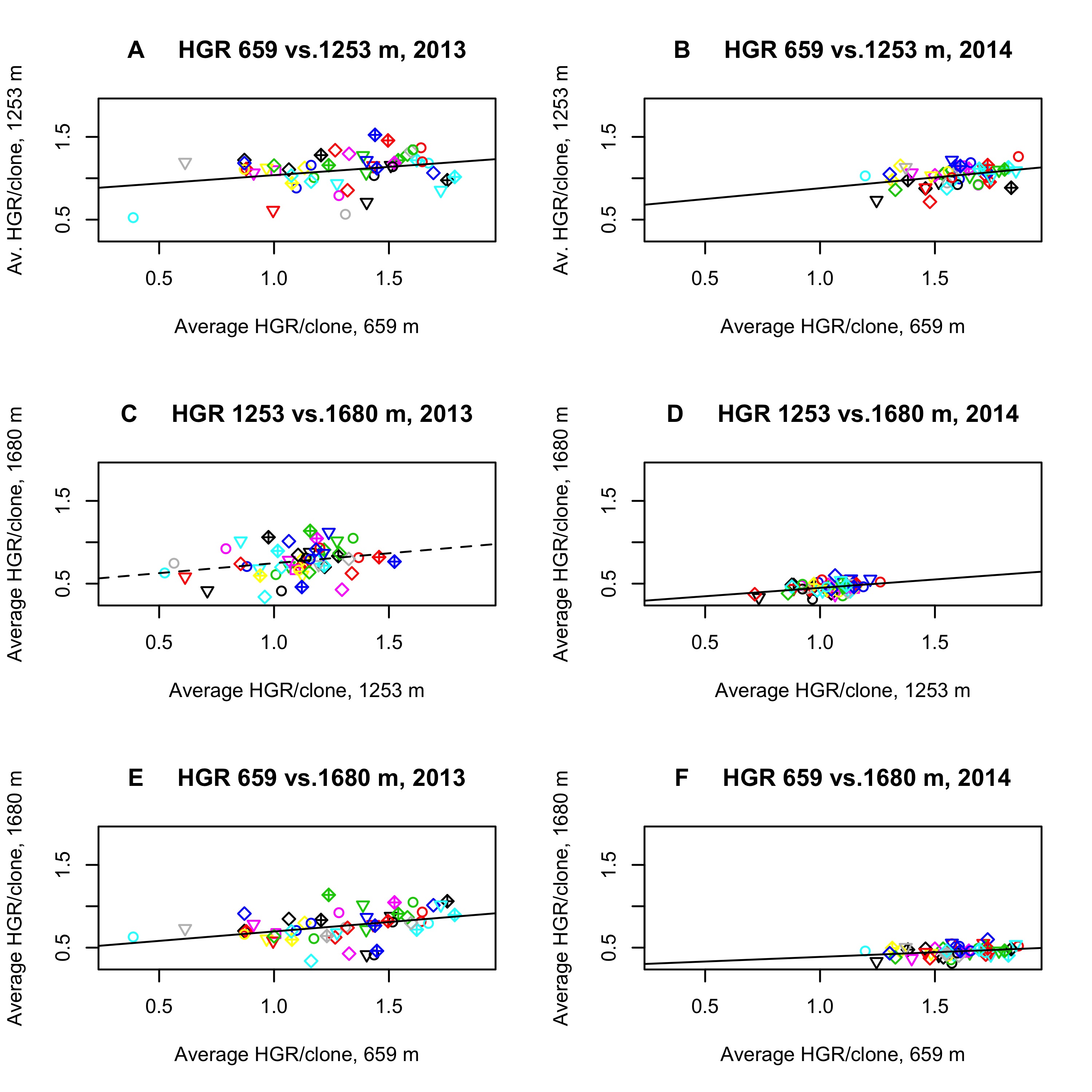


**Figure F:** Correlation in average relative growth rate of each clone across sites in 2013 (left column) and 2014 (right column). Solid lines – significant at 0.05 level. Dashed line – significant at p= 0.1. Colors indicate home population, shapes clone within population. HGR for higher sites looks lower in 2014 than in 2013 because there was a greater difference in the start of the growing season across sites in 2014. However, relative to start of growth in within site, HGR was similar in both years.


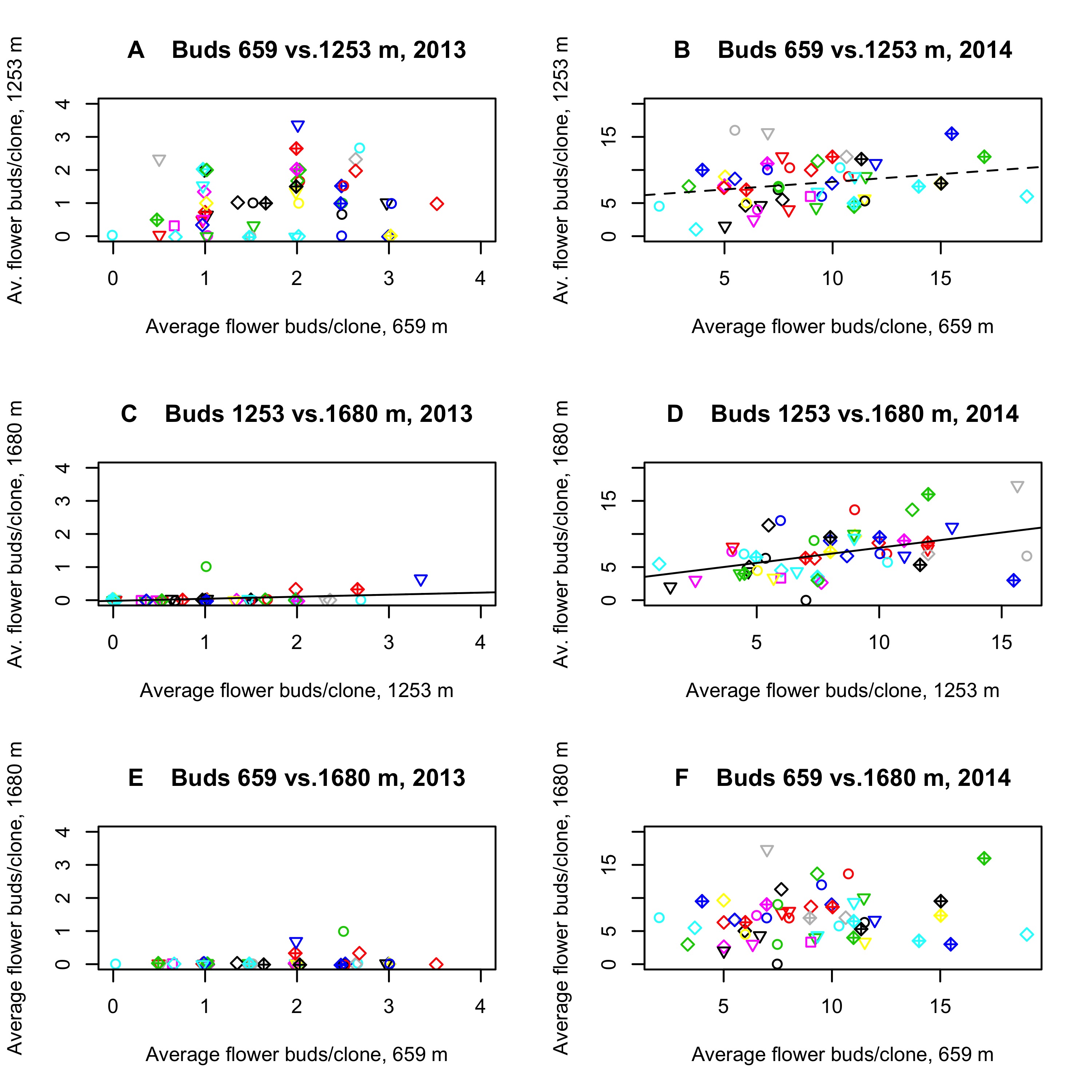


**Figure G:** Correlation in inflorescence bud production of each clone across sites in 2013 (left column) and 2014 (right column). Solid lines – significant at 0.05 level. Dashed line – significant at p=0.1. Notice that the only cross-site comparison that is significant in both years is between the two high-elevation sites. Colors indicate home population, shapes clone within population.

**
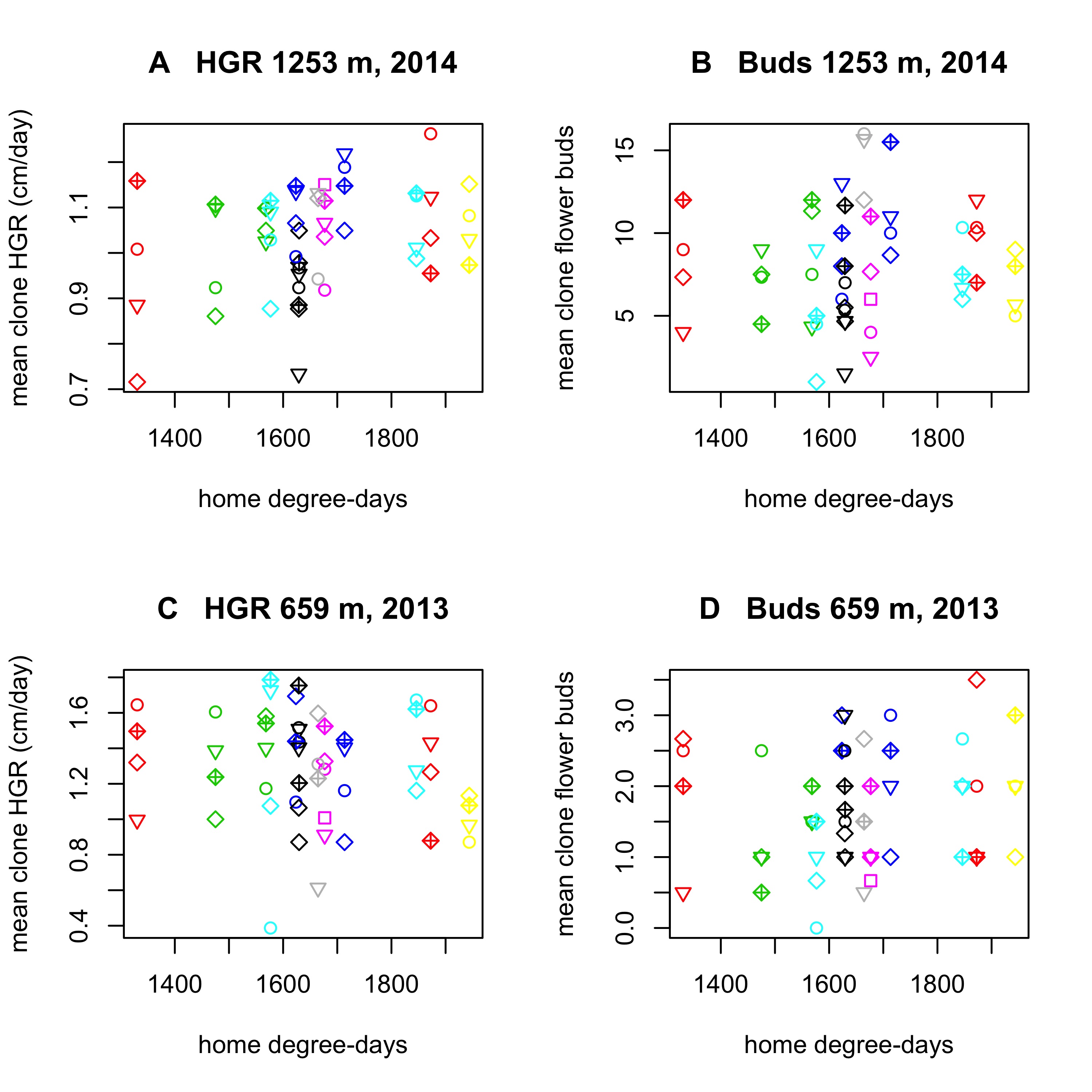
**

**Figure H:** Average height growth rate (left column) or inflorescence buds (right column) by clone. Colors indicate different populations, shapes different clones. Note the wide range in average clone performance within populations.
