# Supplementary material for: Population genetics and adaptation to climate along elevation gradients in invasive Solidago canadensis
Source: PLoS One. 2017 Sep 28;12(9):e0185539. doi: 10.1371/journal.pone.0185539 (PMC5619793; doi:10.1371/journal.pone.0185539)
Supplement: S6 File — (DOCX) [file pone.0185539.s008.docx]

**S6 File: All Bayesian Models Tested**

Green - best model; blue - roughly equivalent models

**Overall best – bold**

Site = Site effects (low, med, high)

Pop = Population ID

Clone = Clone effect (C and S the clone parameters)

Mass = Initial mass

Rhiz = Initial # of rhizome buds

Size = Mass+Rhiz

Env dist = Overall environmental distance based on PCA analysis

Env var = Difference between home and planting site for all environmental variables [DD +P+JT+F+YRAD+Mrad+PF+A+S]

DD = Degree day difference from home site

P = Average monthly precipitation difference from home site

JT = July temperature difference from home site

Frost = Frost index difference from home site

Yrad = Yearly solar radiation difference from home site

Mrad = March solar radiation difference from home site

PF = Average summer rain events difference from home site

A = Aspect difference from home site

Sl = Slope difference from home site

HGR = Height growth rate

**Total Survival:**

| **Model** | **Dm** |
| --- | --- |
| S0 | 100.8 |
| S1 – site | 98.8 |
| S2 – site + env dist | 97.3 |
| S3 – site + env var | 93.3 |
| S4 – env var | 93.1 |
| S5 – site + size | 94.6 |
| S6 – site + env dist + size | 94.7 |
| S7 – size | 100.0 |
| S8 – site + pop | 95.1 |
| S9 – site + size plus a) DD, b) P, c) JT, d) F, e) Yrad, f) Mrad, g) PF, h) Sl, *or* i) A | a)96.7, b)92.1, c)95, d)92.4, e)96.8, f)96.9, g)94.4, h)95.6, i)92.1 |
| S10 – env var + size | 90.2 |
| S11 – clone | 100.8 |
| S12 – site + clone | 96.2 |
| S13 – env var + clone | 91.8 |
| S14 – env dist+ clone | 99.3 |
| S15 – env var + size + clone | 89.2 |
| S16 – size + clone | 97.7 |
| S17 - site + clim var + size | 89.7 |
| S18 - site + size + a) JT&P, b) JT&PF, c) DD & P, d) DD&PF, e) F&P, f) F&PF | a) 91.6, b) 93.1, c) 92.2, d) 93.2, e) 91.1, f) 91.0 |
| S19 - site + size + a) JT&P&YR, b) JT&F&P, c) F&PF&YR d) DD&F&YR | 92.16, 89.946, 89.95, 90.95 |
| S20 - site + clim var + size + clone1 | 88.5 |

**Relative height growth:**

| **Model** | **Dm 2013** | **Dm 2014** |
| --- | --- | --- |
| G0 | 114.5 | 167.2 |
| G1 – site | 81 | 21.3 |
| G2 – site + env dist | 80.9 | 21.1 |
| G3 – site + env var | 79.9 | 20.4 |
| G4 – env var | 80.2 | 22.0 |
| G5 – site + size | 80.0 | 21.5 |
| G6 – site + pop | 80.1 | 20.6 |
| G7 – site + env var + size | 78.5 | 20.5 |
| G8 – site + size+ env var minus a) DD, b) P, c) JT, d) F, e) Yrad, f) Mrad, g) PF, h) Sl, i) A, j) rhiz, or k)mass | a) 78.6, b) 78.3, c) 78.4, d) 78.2, e) 78.8, f) 78.8, g) 78.3, h) 78.1, i) 77.9, j) 78.7, k) 80, | a) 20.5, b) 20.5, c) 20.58, d) 20.5, e) 20.7, f) 20.7, g) 20.5, h) 20.5, i) 20.4, j) 20.4, k) 20.6 |
| G9 – clone | 106.3 | 169.4 |
| G10 – site + clone | 71.6 | 19.1 |
| G11 – env var + clone | 71.7 | 19.2 |
| G12 – site + env var + clone | 71.8 | 19.0 |
| G13 – site + clone + size | 69.6 | 18.9 |
| G14 – site + size+ env var minus a)A&Sl&F, b)A&Sl&PF, or c)A&JT&PF | a) 77.6, b) 78.7, c) 79.1 | a) 20.4, b) 20.5, c)20.9 |
| G15 - site + clone1 + a) rhiz or b)mass] | a)71. 7, b) 70.1 | a) 19.1, b) 19 |
| G16 – site + clone1 + size + a) Clim dist, b) DD, c) precip, d) JT, e) frost, f) Yrad, g) Mrad, h) PrFrq, i) slope, j) aspect | a)69.7, b)70, c 69.8, d)70.1, e)69.9, f)69.7, g)69.9, h)70.1, i)69.8, j)70.1 | a)18.9, b)18.8, c)18.9, d)18.9, e)18.9, f)19, g)18.9, h)18.9, i)18.9, j)18.9 |
| G17 – site + clim var + size + clone1 | 69.7 | 19.0 |

**Flowering:**

| **Model** | **Dm 2013**  **[Total/ Mature]** | **Dm 2014**  **[Total/ Mature]** |
| --- | --- | --- |
| F0 | 1,158.3 / 1,127.7 | 17,638.8 / 6,894,906 |
| F1 – site | 751.4 / 332.2 | 17,547.2 / 15,197.9 |
| F2 – env var | 770.3 / 355.1 | 17,577.2 / 37,912.1 |
| F3 - HGR | 808.0 / 475.7 | 16,373.8 / 30,944.7 |
| F4 - size | 1,134.8 / 823.2 | 16,133.9 / 20,538,470 |
| F5 – site + env dist | 750.8 / 333.1 | 17,512 / 16,298.9 |
| F6 – site + env var | 757.2 / 335.6 | 17,166.8 / 21,653.1 |
| F7 – site + HGR | 689.2 / 294.4 | 14,141.1 / 13,329.8 |
| F8 – site + pop | 751.9 / 330.2 | 17,103.0 / 22,089.0 |
| F9 - clone | 1,158.8 / 1,081.8 | 14,960.3 / 53,788,144 |
| F10 – site +clone | 731.4 /323.0 | 14,653.5 / 14,882.5 |
| F11 - HGR + clone | 781.1 / 455.6 | 13,859.5 / 26,453.5 |
| F12 – env dist + clone | 807.9 / 411.2 | 14,436.9 / 55,023.2 |
| F13 – env var + clone | 752.6/ 349.8 | 14,742.6 / 30,268.9 |
| F14 – env var +HGR | 704.3 /327 | 14,745.6 / 25,487.1 |
| F15 – site + env var + HGR | 682.9 / 302.6 | 13,639.3 / 17,626.5 |
| F16 – site + HGR+ either JT&P, JT&PF, DD & P, or DD&PF | a)702.1, b)692.7, c)697.11, d)693.0  **/** a)300.3, b)302.9, c)302, d)297.5 | a)14,213.1, b)13,938.8, c)14,181, d)14,049.8  **/** a)15,004.8, b)15,706.4, c)15,154.4, d)15,360.9 |
| F17 – env var + HGR + clone | 693.4 / 324.6 | 11,879.5 / 24,569.0 |
| F18 – site + env var + HGR + clone | 677.2 / 299.5 | 11,798.4 /16,369.9 |
| F19 – site + pop +HGR | 683.3 / 299.6 | 13,155.6 / 18,053.6 |
| F20 – size + env var + HGR | 700.1 / 348.1 | 12,777.5 */* 21,555.2 |
| F21 – size + env var + HGR + clone | 672.6 / 316.2 | 10,795.5 / 19,488.9 |
| F22 – site +size + env var + HGR + clone | 660 / 292 | 10,782.2 / 13,904.7 |
