# Supplementary material for: Population genetics and adaptation to climate along elevation gradients in invasive Solidago canadensis
Source: PLoS One. 2017 Sep 28;12(9):e0185539. doi: 10.1371/journal.pone.0185539 (PMC5619793; doi:10.1371/journal.pone.0185539)
Supplement: S7 File — (DOCX) [file pone.0185539.s009.docx]

S7 File: Best-fitting survival models.

Because Dm calculation can vary by up to 3 between model runs, fits for the models included on this chart are roughly equivalent. Estimated parameter values, rounded to a few decimal points, are shown. ~0 indicates that the parameter value was < 0.005. Int- intercept of GLM. L/M/H - low, medium, and high garden; Environmental distance DD/P/JT/F/YR/MR/PF/Sl/A = degree day, precipitation, July temperature, frost index, annual and march radiation, slope, and aspect difference of planting site from home site. Size RB/M - initial rhizome buds and mass. C - mean clone effect. S- variance in clone effects. However, please note that, because some groups of environmental variables are strongly correlated (DD, JT, & F; P & PF; YR & MR) there can be a tendency for tradeoffs in parameter estimation and so the strength and direction of these effects individually should not be taken at face value.

|  |  |  | **Site** | | | **Environmental distance** | | | | | | | | | **Size** | | **Clone Eff.** | |  |
| --- | --- | --- | --- | --- | --- | --- | --- | --- | --- | --- | --- | --- | --- | --- | --- | --- | --- | --- | --- |
|  | **Dm** | **Int** | **L** | **M** | **H** | **DD** | **P** | **JT** | **F** | **YR** | **MR** | **PF** | **Sl** | **A** | **RB** | **M** | **C** | **S** | **Sig** |
| **Model** |  |  |  |  |  |  |  |  |  |  |  |  |  |  |  |  |  |  |  |
| **S20** | 88.5 | **1.7** | -0.3 | -0.3 | 0.3 | -0.06 | -0.06 | 0.04 | 0.05 | -0.15 | 0.05 | ~0 | -0.03 | ~0 | 0.10 | 0.18 | -0.08 | 0.09 | 0.2 |
| **S15** | 89.2 | **1.7** |  |  |  | -0.04 | -0.04 | -0.07 | 0.05 | -0.08 | -0.03 | ~0 | -0.03 | ~0 | 0.10 | 0.17 | -0.21 | 0.09 | 0.5 |
| **S17** | 89.7 | **1.6** | -0.3 | -0.3 | 0.3 | -0.06 | -0.07 | 0.04 | 0.05 | -0.16 | 0.07 | 0.01 | -0.02 | ~0 | 0.09 | 0.15 |  |  | 0.2 |
| **S19b** | 89.95 | **1.1** | -0.6 | -0.4 | 0.3 |  | -0.15 | -0.08 | 0.05 |  |  |  |  |  | 0.08 | 0.16 |  |  | 0.1 |
| **S19c** | 89.95 | **1.5** | -0.6 | -0.3 | 0.5 |  |  |  | 0.07 | -0.07 |  | -0.05 |  |  | 0.07 | 0.16 |  |  | 0.2 |
| **S10** | 90.2 | **1.5** |  |  |  | 0.04 | -0.02 | -0.19 | 0.04 | -0.08 | -0.01 | -0.02 | -0.03 | ~0 | 0.10 | 0.15 |  |  | 0.2 |
| **S19d** | 90.95 | **1.4** | -0.7 | -0.2 | 0.4 | 0.01 |  |  | 0.08 | -0.16 |  |  |  |  | 0.08 | 0.13 |  |  | 0.2 |
| **S18f** | 91.0 | **1.5** | -0.6 | -0.3 | 0.4 |  |  |  | 0.05 |  |  | -0.05 |  |  | 0.07 | 0.17 |  |  | 0.2 |
| **S18e** | 91.1 | **1.3** | -0.7 | -0.4 | 0.6 |  | -0.12 |  | 0.05 |  |  |  |  |  | 0.1 | 0.16 |  |  | 0.3 |
| **S18a** | 91.5 | **1.2** | -0.7 | -0.5 | 0.7 |  | -0.17 | -0.12 |  |  |  |  |  |  | 0.08 | 0.13 |  |  | 0.2 |

Predictor variable ranges and units:

DD: -14.52 to 1.99 degrees*days/100

P: -6.07 to 7.96 cm

JT: -8.03 to 0.67 °C

F: -22 to 18.42 frost index units

YR: -2 to 8.17 MJ/m^2^/day

MR: 3 to 24.3 MJ/m^2^/day

PF: -7 to 22 days

Sl: -14.6 to 24.6 degrees inclination

A: -283 to 135.9 compass degrees

RB: 1 to 20 buds

M: 0.5 to 26 grams
