# Supplementary material for: Population genetics and adaptation to climate along elevation gradients in invasive Solidago canadensis
Source: PLoS One. 2017 Sep 28;12(9):e0185539. doi: 10.1371/journal.pone.0185539 (PMC5619793; doi:10.1371/journal.pone.0185539)
Supplement: S8 File — (DOCX) [file pone.0185539.s010.docx]

S8 File: Best-fitting relative height-growth models

Because Dm calculation can vary by up to 1.2 between model runs, fits for the models included on this chart are considered roughly equivalent. In fact, all G16 models were roughly equivalent, so only a subset of these single-environmental-distance models are shown here.

Estimated parameter values, rounded to a few decimal points, are shown. ~0 indicates that the parameter value was < 0.005. Int- intercept of GLM. L/M/H - low, medium, and high garden; Environmental distance DD/P/JT/F/YR/MR/PF/Sl/A = degree day, precipitation, July temperature, frost index, annual and march radiation, slope, and aspect difference of planting site from home site. Size RB/M - initial rhizome buds and mass. C - mean clone effect. S- variance in clone effects. However, please note that, because some groups of environmental variables are strongly correlated (DD, JT, & F; P & PF; YR & MR) there can be a tendency for tradeoffs in parameter estimation and so the strength and direction of these effects individually should not be taken at face value.

|  |  |  | **Site** | | | **Environmental distance** | | | | | | | | | **Size** | | **Clone Eff.** | |  |
| --- | --- | --- | --- | --- | --- | --- | --- | --- | --- | --- | --- | --- | --- | --- | --- | --- | --- | --- | --- |
|  | **Dm** | **Int** | **L** | **M** | **H** | **DD** | **P** | **JT** | **F** | **YR** | **MR** | **PF** | **Sl** | **A** | **RB** | **M** | **C** | **S** | **Sig** |
| **HGR'13** |  |  |  |  |  |  |  |  |  |  |  |  |  |  |  |  |  |  |  |
| **G13** | 69.6 | 1.2 | 0.3 | 0.1 | -0.3 |  |  |  |  |  |  |  |  |  | -0.01 | 0.02 | -0.28 | 0.01 | 0.1 |
| **G16f** | 69.7 | 0.7 | 0.2 | 0.02 | -0.3 |  |  |  |  |  | ~0 |  |  |  | -0.01 | 0.02 | 0.32 | 0.01 | 0.1 |
| **G16a** | 69.7 | 1.1 | 0.05 | 0.07 | -0.2 | 0.03 |  |  |  |  |  |  |  |  | -0.01 | 0.02 | 0.15 | 0.01 | 0.1 |
| **G17** | 69.7 | 1.1 | ~0 | 0.11 | -0.2 | ~0 | 0.02 | 0.06 | ~0 | 0.08 | -0.06 | -0.02 | ~0 | ~0 | -0.01 | 0.02 | 0.2 | 0.02 | 0.1 |
| **G16i** | 69.8 | 1.1 | 0.2 | 0.1 | -0.3 |  |  |  |  |  |  |  | ~0 |  | -0.01 | 0.02 | -0.07 | 0.01 | 0.1 |
| **G16c** | 69.8 | 1.3 | 0.12 | 0.17 | -0.1 |  |  | 0.06 |  |  |  |  |  |  | -0.01 | 0.02 | -0.18 | 0.01 | 0.1 |
| **G15b** | 70.1 | 1 | 0.2 | 0.1 | -0.3 |  |  |  |  |  |  |  |  |  |  | 0.01 | -0.03 | 0.01 | 0.1 |
| **HGR'14** |  |  |  |  |  |  |  |  |  |  |  |  |  |  |  |  |  |  |  |
| **G16b** | 18.8 | 0.9 | 0.5 | -0.02 | -0.6 |  | 0.01 |  |  |  |  |  |  |  | ~0 | 0.01 | 0.12 | 0.01 | 0.03 |
| **G16h** | 18.8 | 1 | 0.2 | 0.07 | -0.3 |  |  |  |  |  |  | ~0 |  |  | -0.01 | 0.02 | 0.04 | 0.01 | 0.03 |
| **G16c** | 18.9 | 1 | 0.6 | ~0 | -0.6 |  |  | -0.01 |  |  |  |  |  |  | ~0 | 0.01 | -0.04 | 0.01 | 0.03 |
| **G16d** | 18.9 | 1.1 | 0.6 | 0.04 | -0.5 |  |  |  | ~0 |  |  |  |  |  | ~0 | 0.01 | -0.1 | 0.01 | 0.03 |
| **G13** | 18.9 | 0.9 | 0.5 | -0.3 | -0.6 |  |  |  |  |  |  |  |  |  | ~0 | 0.01 | 0.15 | 0.01 | 0.03 |
| **G15b** | 19 | 1.1 | 0.6 | 0.01 | -0.6 |  |  |  |  |  |  |  |  |  |  | 0.01 | -0.08 | 0.01 | 0.02 |
| **G12** | 19 | 1.3 | 0.5 | 0.2 | -0.3 | -0.09 | 0.03 | 0.2 | ~0 | 0.06 | -0.06 | -0.01 | ~0 | ~0 |  |  | -0.8 | 0.02 | 0.03 |
| **G17** | 19 | 1.2 | 0.4 | 0.1 | -0.4 | -0.08 | 0.03 | 0.19 | ~0 | 0.06 | -0.06 | -0.01 | ~0 | ~0 | ~0 | 0.01 | 0.11 | 0.02 | 0.03 |
| **G10** | 19.1 | 0.9 | 0.2 | 0.05 | -0.3 |  |  |  |  |  |  |  |  |  |  |  | 0.12 | 0.01 | 0.03 |
| **G15a** | 19.1 | 1 | 0.5 | ~0 | -0.6 |  |  |  |  |  |  |  |  |  | ~0 |  | 0.07 | 0.01 | 0.03 |
| **G11** | 19.2 | 1.3 |  |  |  | -0.33 | -0.01 | 0.74 | ~0 | 0.02 | -0.04 | 0.02 | ~0 | ~0 |  |  | 0.36 | 0.02 | 0.03 |

Predictor variable ranges and units:

DD: -14.52 to 1.99 degrees*days/100

P: -6.07 to 7.96 cm

JT: -8.03 to 0.67 °C

F: -22 to 18.42 frost index units

YR: -2 to 8.17 MJ/m^2^/day

MR: 3 to 24.3 MJ/m^2^/day

PF: -7 to 22 days

Sl: -14.6 to 24.6 degrees inclination

A: -283 to 135.9 compass degrees

RB: 1 to 20 buds

M: 0.5 to 26 grams
