# Supplementary material for: Population genetics and adaptation to climate along elevation gradients in invasive Solidago canadensis
Source: PLoS One. 2017 Sep 28;12(9):e0185539. doi: 10.1371/journal.pone.0185539 (PMC5619793; doi:10.1371/journal.pone.0185539)
Supplement: S9 File — (DOCX) [file pone.0185539.s011.docx]

S9 File: Best-fitting flowering models

Because Dm calculation can vary by up to 20 or 100 for total flowers, and by 10 or 580 for mature flowers in 2013 & 2014, respectively, between model runs, fits for the models shown are roughly equivalent.

Estimated parameter values, rounded to a few decimal points, are shown. ~0 indicates that the parameter value was < 0.005. Int- intercept of GLM. L/M/H - low, medium, and high garden; Environmental distance DD/P/JT/F/YR/MR/PF/Sl/A = degree day, precipitation, July temperature, frost index, annual and march radiation, slope, and aspect difference of planting site from home site. Size RB/M - initial rhizome buds and mass. C - mean clone effect. S- variance in clone effects. However, please note that, because some groups of environmental variables are strongly correlated (DD, JT, & F; P & PF; YR & MR) there can be a tendency for tradeoffs in parameter estimation and so the strength and direction of these effects individually should not be taken at face value.

Table 1: Total flower buds

|  |  |  | **Site** | | | **Environmental distance** | | | | | | | | | **Size** | | **Clone Eff.** | |  |  |
| --- | --- | --- | --- | --- | --- | --- | --- | --- | --- | --- | --- | --- | --- | --- | --- | --- | --- | --- | --- | --- |
|  | **Dm** | **Int** | **L** | **M** | **H** | **DD** | **P** | **JT** | **F** | **YR** | **MR** | **PF** | **Sl** | **A** | **RB** | **M** | **C** | **S** | **HGR** | **Sig** |
| **TF'13** |  |  |  |  |  |  |  |  |  |  |  |  |  |  |  |  |  |  |  |  |
| F22 | 660 | -1 | -0.4 | -0.5 | -2.8 | -0.15 | -0.09 | 0.27 | -0.02 | 0.08 | -0.05 | 0.05 | 0.01 | ~0 | 0.03 | 0.05 | -0.7 | 0.07 | 1.4 | 0.05 |
| F21 | 673 | -1.8 |  |  |  | -0.8 | -0.3 | 1.68 | -0.01 | -0.2 | 0.22 | 0.2 | 0.03 | ~0 | 0.03 | 0.04 | -1.1 | 0.07 | 1.4 | 0.05 |
| F18 | 677 | -0.9 | -0.4 | -0.5 | -2.7 | -0.28 | -0.13 | 0.52 | -0.01 | ~0 | -0.02 | 0.08 | 0.01 | ~0 |  |  | -0.6 | 0.05 | 1.4 | 0.05 |
| **TF'14** |  |  |  |  |  |  |  |  |  |  |  |  |  |  |  |  |  |  |  |  |
| F22 | 10,782 | -0.8 | -0.9 | -0.6 | -0.2 | 0.39 | -0.1 | -0.93 | ~0 | -0.08 | 0.1 | 0.01 | ~0 | ~0 | 0.02 | 0.04 | 0.03 | 0.05 | 1.9 | 0.1 |
| F21 | 10,796 | 0.2 |  |  |  | 0.52 | -0.03 | -1.22 | -0.01 | -0.02 | 0.05 | -0.02 | ~0 | ~0 | 0.02 | 0.04 | -1.4 | 0.06 | 1.7 | 0.1 |

Table 2: Mature flowers

|  |  |  | **Site** | | | **Environmental distance** | | | | | | | | | **Size** | | **Clone Eff.** | |  |  |
| --- | --- | --- | --- | --- | --- | --- | --- | --- | --- | --- | --- | --- | --- | --- | --- | --- | --- | --- | --- | --- |
|  | **Dm** | **Int** | **L** | **M** | **H** | **DD** | **P** | **JT** | **F** | **YR** | **MR** | **PF** | **Sl** | **A** | **RB** | **M** | **C** | **S** | **HGR** | **Sig** |
| **MF'13** |  |  |  |  |  |  |  |  |  |  |  |  |  |  |  |  |  |  |  |  |
| F22 | 292 | -2.3 | 1.1 | -6.5 | -6 | -0.13 | -0.03 | 0.32 | ~0 | -0.03 | -0.03 | 0.03 | -0.01 | ~0 | 0.03 | 0.08 | -0.8 | 0.06 | 1.2 | 0.05 |
| F16d | 297.5 | -2.6 | 1.1 | -6.1 | -6.7 | ~0 |  |  |  |  |  | 0.01 |  |  |  |  |  |  | 1.2 | 0.1 |
| F18 | 299.5 | -2.2 | 1.2 | -6.2 | -6.2 | -0.4 | -0.13 | 0.76 | 0.01 | -0.18 | 0.05 | 0.1 | ~0 | ~0 |  |  | -1 | 0.06 | 1.3 | 0.1 |
| F19 | 299.6 | -1.2 | -0.3 | -0.4 | -3 |  |  |  |  |  |  |  |  |  |  |  |  |  | 1.4 | 0.05 |
| F16a | 301.1 | -2.0 | 0.5 | -5.8 | -5.8 |  | -0.03 | 0.01 |  |  |  |  |  |  |  |  |  |  | 1.2 | 0.1 |
| **MF'14** |  |  |  |  |  |  |  |  |  |  |  |  |  |  |  |  |  |  |  |  |
| F7 | 13,330 | -2.3 | 2.3 | 0.6 | -6.1 |  |  |  |  |  |  |  |  |  |  |  |  |  | 1.3 |  |
| F22 | 13,905 | -2 | 2 | 1 | -6 | 0.24 | -0.05 | -0.36 | ~0 | 0.32 | -0.22 | -0.02 | ~0 | ~0 | -0.01 | 0.1 | -0.7 | 0.06 | 1.5 | 0.4 |

Population effect estimates from F19 (Mature flowers, 2013):

LM10 (276 m): 0.05

LH6 (449 m): 0.30

LH1 (450 m): -0.42

LH2 (452 m): 0.20

LL1 (475 m): -0.11

LM2 (543 m): -0.16

LM6 (544 m): -0.04

LH9 (569 m): -0.07

LL2 (600 m): -0.13

ML7 (661 m): -0.21

MH1 (667 m): 0.35

ML6 (782 m): 0.22

HL1 (816 m): -0.66

Predictor variable ranges and units:

DD: -14.52 to 1.99 degrees*days/100

P: -6.07 to 7.96 cm

JT: -8.03 to 0.67 °C

F: -22 to 18.42 frost index units

YR: -2 to 8.17 MJ/m^2^/day

MR: 3 to 24.3 MJ/m^2^/day

PF: -7 to 22 days

Sl: -14.6 to 24.6 degrees inclination

A: -283 to 135.9 compass degrees

RB: 1 to 20 buds

M: 0.5 to 26 grams
